# Unraveling the functions of uncharacterized transcription factors in *Escherichia coli* using ChIP-exo

**DOI:** 10.1093/nar/gkab735

**Published:** 2021-08-24

**Authors:** Ye Gao, Hyun Gyu Lim, Hans Verkler, Richard Szubin, Daniel Quach, Irina Rodionova, Ke Chen, James T Yurkovich, Byung-Kwan Cho, Bernhard O Palsson

**Affiliations:** Department of Biological Sciences, University of California San Diego, La Jolla, CA 92093, USA; Department of Bioengineering, University of California San Diego, La Jolla, CA 92093, USA; Department of Bioengineering, University of California San Diego, La Jolla, CA 92093, USA; Department of Bioengineering, University of California San Diego, La Jolla, CA 92093, USA; Department of Bioengineering, University of California San Diego, La Jolla, CA 92093, USA; Department of Biological Sciences, University of California San Diego, La Jolla, CA 92093, USA; Department of Bioengineering, University of California San Diego, La Jolla, CA 92093, USA; Department of Bioengineering, University of California San Diego, La Jolla, CA 92093, USA; Department of Bioengineering, University of California San Diego, La Jolla, CA 92093, USA; Department of Bioengineering, University of California San Diego, La Jolla, CA 92093, USA; Department of Biological Sciences and KI for the BioCentury, Korea Advanced Institute of Science and Technology, Daejeon 34141, Republic of Korea; Department of Bioengineering, University of California San Diego, La Jolla, CA 92093, USA; Department of Pediatrics, University of California San Diego, La Jolla, CA 92093, USA; Bioinformatics and Systems Biology Program, University of California San Diego, La Jolla, CA 92093, USA; Novo Nordisk Foundation Center for Biosustainability, 2800, Kongens Lyngby, Denmark

## Abstract

Bacteria regulate gene expression to adapt to changing environments through transcriptional regulatory networks (TRNs). Although extensively studied, no TRN is fully characterized since the identity and activity of all the transcriptional regulators comprising a TRN are not known. Here, we experimentally evaluate 40 uncharacterized proteins in *Escherichia coli* K-12 MG1655, which were computationally predicted to be transcription factors (TFs). First, we used a multiplexed chromatin immunoprecipitation method combined with lambda exonuclease digestion (multiplexed ChIP-exo) assay to characterize binding sites for these candidate TFs; 34 of them were found to be DNA-binding proteins. We then compared the relative location between binding sites and RNA polymerase (RNAP). We found 48% (283/588) overlap between the TFs and RNAP. Finally, we used these data to infer potential functions for 10 of the 34 TFs with validated DNA binding sites and consensus binding motifs. Taken together, this study: (i) significantly expands the number of confirmed TFs to 276, close to the estimated total of about 280 TFs; (ii) provides putative functions for the newly discovered TFs and (iii) confirms the functions of four representative TFs through mutant phenotypes.

## INTRODUCTION

Bacteria employ a broad range of mechanisms to regulate gene expression to achieve and maintain phenotypic states (1). The primary mechanism by which gene expression is regulated in bacteria relies on the promoter recognition by the RNA polymerase (RNAP) holoenzyme and its subsequent initiation of transcription (2). Since the core enzyme (including α, α, β, β’ and ω) itself is unable to recognize promoters or to initiate transcription, a sigma factor, which directly recognizes its target sequence, binds to the core enzyme, forming a complex known as the RNA polymerase holoenzyme. This complex then orchestrates transcription initiation from specific promoters (1). In addition to the regulation by sigma factors, transcription factors (TFs) also bind to intergenic regulatory regions of DNA, preventing or promoting RNAP binding upstream from a transcription start site (3). Thus, the identification of transcription factors and their association with sigma factors is fundamental to understanding how an organism responds to varying phenotypic demands through transcriptional regulation.

A complete description of the *Escherichia coli* K-12 transcriptional regulatory network (TRN) is of particular importance to the scientific community because it provides fundamental information not only for unravelling regulatory network architectures that are host to individual regulators and their target genes, but also for studying the interactions among multiple regulators. Although *E. coli* K-12 MG1655 is one of the best understood model organisms, our current knowledge of its TRN is still incomplete (4,5). To reconstruct the global TRN, it is necessary to identify a full set of TFs and expand the TRN through new chromatin immunoprecipitation (ChIP) data for individual TFs.

We previously developed a pipeline for computational prediction followed by experimental validation via ChIP technology (6,7). The first use of this pipeline successfully discovered ten novel TFs in *E. coli* and identified their regulatory roles. In this study, to get closer to the complete characterization of the *E. coli* K-12 MG1655 TRN, we employ this pipeline again to characterize an additional 40 candidate TFs and their target genes. Specifically, we use a high-throughput method (multiplexed ChIP-exo) to generate massive protein-DNA interactions datasets for these candidate TFs, RNAP, and the sigma factor RpoD. Combining these data, we successfully uncover 588 binding sites of 34 TFs from 40 initial candidates, in which 283 binding sites are located upstream. Based on the number of target genes, we classify these TFs into three groups: (i) one global regulator (>100 target genes), (ii) twenty-nine local regulators (<100 target genes) and (iii) four single-target regulators (8). We further explore the physiological roles of four representative TFs using gene expression profiling and mutant phenotype analysis. Our results illustrate that newly discovered TFs have a varied number of regulatory targets and participate in key cellular processes from replication, transcription, nutrition metabolism to stress responses in *E. coli* K-12 MG1655. Taken together, our results expand the total number of validated TFs to 276 (an increase of ∼12%), and support the estimated total of 280∼300 TFs comprising the TRN in *E. coli* K-12 MG1655 (9).

## MATERIALS AND METHODS

### Computational prediction of candidate TFs

Previously, we had generated a list of candidate TFs and used 16 of the top candidates to assess the discovery pipeline (10). Ten of the 16 candidates were found to be TFs. Here, we extended the experimental validation of these computationally predicted targets by selecting and studying additional candidates from this previous list. Briefly, the list was generated using the TFpredict algorithm (11) modified for use with bacterial genomes (10). The TFpredict algorithm takes a protein sequence as input and generates a quantified score in the range [0,1] that represents the likelihood of that protein being a TF based on sequence homology, where a score of 1 represents the highest confidence. We selected 40 of the top candidate TFs from this rank-ordered list. See reference (10) for a full description of the computational methods.

### Bacterial strains, media and growth conditions

The strains used in this study are *E. coli* K-12 MG1655 and its derivatives, deletion strains, and myc-tagged strains (Dataset S1). For ChIP-exo experiments, the *E. coli* strains harboring 8-myc were generated by a λ red-mediated site-specific recombination system targeting the C-terminal region as described previously (12). For ChIP-exo experiments, glycerol stocks of *E. coli* strains were inoculated into M9 minimal medium (47.8 mM Na_2_HPO_4_, 22 mM KH_2_PO_4_, 8.6 mM NaCl, 18.7 mM NH_4_Cl, 2 mM MgSO_4_ and 0.1 mM CaCl_2_) with 0.2% (w/v) glucose. The M9 minimal medium was also supplemented with 1 ml trace element solution (100X) containing 1 g EDTA, 29 mg ZnSO_4_.7H_2_O, 198 mg MnCl_2_.4H_2_O, 254 mg CoCl_2_.6H_2_O, 13.4 mg CuCl_2_ and 147 mg CaCl_2_. The culture was incubated at 37°C overnight with agitation and was then used to inoculate fresh media (1/200 dilution). The volume of the fresh media was 150 mL per biological replicate. The fresh culture was incubated at 37°C with agitation to the mid-log phase where optimal density at 600 nm (OD_600_) was around 0.5. To create oxidative stress, the overnight cultures were inoculated at an OD_600_ of 0.01 into the fresh 70 mL of glucose M9 minimal medium in a 500 ml flask supplemented with 250 μM paraquat (PQ) at an OD_600_ of 0.3 and incubated for 20 min with stirring. The strains in the ChIP-exo experiments were grown under the conditions listed in Dataset S2.

To evaluate the susceptibility of bacterial cells to H_2_O_2_, mid-log phase cells (OD_600_ ≈ 0.5) were harvested, washed with phosphate-buffered saline (PBS), and resuspended in M9 minimal medium. The culture was then treated with 60 mM H_2_O_2_ (the final concentration) for 15 min. Samples were taken before and after the treatment, diluted, and plated in triplicate on LB plates. Viable counts were determined following incubation at 37°C for up to 24 h. The sensitivity of cells to the lethal effect of the stimulus was expressed as percent survival of treated cells relative to that of untreated cells determined at time zero.

To examine the effects of carbon sources on cell growth, *E. coli* K-12 MG1655 and *yciT* deletion strains were incubated on M9 minimal medium with a sole carbon source (glucose, fructose, or sorbitol) at 37°C overnight with agitation. The concentration of the carbon sources was 0.2% (w/v). These cultures were then used to inoculate the same fresh media (1/200 dilution) and were incubated again at 37°C with agitation. Growth curves were monitored by measuring OD_600_ every 30 min using a Bioscreen C (Growth curves, USA), and repeated twice with three biological replicates.

To determine the effects of osmotic stress on the growth, *E. coli* K-12 MG1655 and *yciT* deletion strains were grown on M9 minimal sorbitol (0.2% w/v) media and the same media supplemented with 0.5 M NaCl at the beginning of the culture, respectively. The culture was incubated at 37°C with agitation and monitored by measuring OD_600_ every 30 min using a Bioscreen C, and repeated twice with three biological replicates.

### Multiplexed ChIP-exo experiment

A multiplexed ChIP-exo experiment was performed through simple modification of our standard ChIP-exo method described previously (13). Here, after ligating the first adapter to each sample separately, the samples are then pooled together and subject to the remainder of the enzymatic reactions used for library preparation. Each sample receives a different first adapter bearing a unique 6-base sequence (barcode), thus allowing demultiplexing of sequencing data.

To identify the binding map of each candidate TF *in vivo*, the DNA bound to each candidate TF from formaldehyde cross-linked *E. coli* cells were isolated by chromatin immunoprecipitation (ChIP) with the antibody that specifically recognizes the myc tag (9E10, Santa Cruz Biotechnology) and Dynabeads Pan Mouse IgG magnetic beads (Invitrogen). This step was followed by stringent washings (14). Cells were initially grown in glucose minimal medium to OD_600_ = 0.5 and incubated with 1% formaldehyde (Thermo Scientific) for 25 min at room temperature. The formaldehyde was quenched by 2.5 M glycine (Thermo Fisher Scientific) for an additional 5 min and the cells were washed with ice-cold TBS (Thermo Fisher Scientific) three times. The resulting pellets were lysed with Ready-lyse lysozyme solution (Epicentre). Lysates were sonicated using a sonicator (QSonic) to generate 300–500 bp randomly sheared chromosomal DNA fragments. The extent of shearing was monitored with a 1% agarose gel and confirmed by separation on a 2100 High sensitivity Bioanalyzer chip (Agilent Technologies) upon completion of the immunoprecipitation. Immunoprecipitation was carried out at 4°C with overnight incubation and 15 μl anti-c-myc mouse antibody (9E10, Santa Cruz Biotechnology). The protein of interest, together with its cross-linked DNA and covalently bound mouse antibody, was captured with 50 μl Dynabeads Pan mouse IgG (Invitrogen) and washed with buffer I (50 mM Tris–HCl (pH 7.5), 140 mM NaCl, 1 mM EDTA, 1% Triton X-100).

ChIP materials (chromatin-beads) were used to perform on-bead enzymatic reactions of the ChIP-exo method (7). The sheared DNA of chromatin-beads was repaired by the NEBNext End Repair Module (New England Biolabs) followed by the addition of a single dA overhang and ligation of a first adaptor (5′-phosphorylated) using the dA-Tailing Module (New England Biolabs) and the NEBNext Quick Ligation Module (New England Biolabs), respectively. The first adaptor was designed to have different indices to distinguish different DNA samples after the sequencing. After ligation, multiple ChIP materials could be pooled together. Nick repair was performed by using PreCR Repair Mix (New England Biolabs). Lambda exonuclease- and RecJ_f_ exonuclease-treated chromatin was eluted from the beads and incubated overnight at 65°C to reverse the protein–DNA cross-link. RNAs- and proteins-removed DNA samples were used to perform primer extension and second adaptor ligation with following modifications. The DNA samples incubated for primer extension as described previously (13) were treated with dA-Tailing Module (New England Biolabs) and NEBNext Quick Ligation Module (New England Biolabs) for second adaptor ligation. The DNA sample purified by GeneRead Size Selection Kit (Qiagen) was enriched by polymerase chain reaction (PCR) using Phusion High-Fidelity DNA Polymerase (New England Biolabs). The amplified DNA samples were purified again by GeneRead Size Selection Kit (Qiagen) and quantified using Qubit dsDNA HS Assay Kit (Life Technologies). Quality of the DNA sample was checked by running Agilent High Sensitivity DNA Kit using Agilent 2100 Bioanalyzer (Agilent) before sequenced using HiSeq 2500 (Illumina) following the manufacturer's instructions. The antibody (NT63, Biolegend) that specifically recognizes RNA polymerase β was used to conduct the ChIP-exo experiment to detect the binding sites of RNA polymerase in *E. coli* K-12 MG1655. The antibody (2G10, Biolegend) that specifically recognizes σ^70^ was used to detect the binding sites of σ^70^ in *E. coli* K-12 MG1655. Each step was also performed following the manufacturer's instructions. ChIP-exo experiments were performed in biological duplicates (Dataset S3 and S4).

### Peak calling for ChIP-exo dataset

Peak calling was performed as previously described (13). Sequence reads generated from ChIP-exo were mapped onto the reference genome (NC_000913.2) using bowtie (15) with default options to generate SAM output files. The MACE program was used to define peak candidates from biological duplicates for each experimental condition with sequence depth normalization (16). To reduce false-positive peaks, peaks with a signal-to-noise (S/N) ratio <1.5 were removed; and peaks without expected bimodal shape were removed (17) The noise level was set to the top 5% of signals at genomic positions (13). The calculation of S/N ratio resembles the way to calculate ChIP-chip peak intensity where the IP signal was divided by Mock signal. Finally, each peak was assigned to the target gene, according to genomic position (Supplementary Figure S1). Genome-scale data were visualized using MetaScope (https://sites.google.com/view/systemskimlab/software?authuser=0) and NimbleGen's SignalMap software.

### Motif search from ChIP-exo peaks

The consensus DNA sequence motif analysis for validated TFs was performed using the MEME software suite (the *E*-value < 1e-3) (18). For YciT, YcjW, YdcN, YdhB, YfeC, YfeD and YidZ, sequences in binding regions were extracted from the reference genome (NC_000913.2).

### COG functional enrichment

Regulon genes were categorized according to their annotated clusters of orthologous groups (COG) category (19). Functional enrichment of COG categories in the target genes was determined by performing a hypergeometric test, and a *P*-value <0.01 was considered significant.

### Transcriptomics

RNA-seq was performed using two biological replicates (Dataset S5). The strains were grown under the same conditions as those used in the ChIP-exo experiments. Transcripts were stabilized by mixing 3 ml of cell cultures at the mid-log phase with 6 ml of RNAprotect Bacteria Reagent (Qiagen). Samples were immediately vortexed for 5 s, incubated for 5 min at room temperature, and then centrifuged at 5000 × *g* for 10 min. The supernatant was decanted, and any residual supernatant was removed by inverting the tube once onto a paper towel. Total RNA samples were then isolated using a RNeasy Plus Mini kit (Qiagen) following the manufacturer's instruction. Samples were then quantified using a NanoDrop 1000 spectrophotometer (Thermo Scientific) and quality of the isolated RNA was checked by running RNA 6000 Pico Kit using an Agilent 2100 Bioanalyzer (Agilent). Paired-end, strand-specific RNA-seq libraries were prepared using KAPA RNA Hyper Prep kit (KAPA Biosystems), following the instructions (20,21). Resulting libraries were analyzed on an Agilent Bioanalyzer DNA 1000 chip (Agilent). Sequencing was performed on a Hiseq 2500 sequencer (illumina) at the Genomics Core facility of University of California, San Diego.

### Calculation of differentially expressed genes

Expression profiling was performed as previously described (13). Raw sequence reads generated from RNA-seq were mapped onto the reference genome (NC_000913.2) using bowtie v1.2.3 with the maximum insert size of 1000 bp, and two maximum mismatches after trimming 3 bp at 3′ ends (15). Transcript abundance was quantified using summarizeOverlaps from the R GenomicAlignments package, with strand inversion for the dUTP protocol and strict intersection mode (22). We then calculated the dispersion and differential expression level of each gene using DESeq2 (23). DESeq2 uses empirical Bayes shrinkage for dispersion estimation which substantially improves the stability and reproducibility of analysis results compared to maximum-likelihood-based solutions. This also makes DESeq2 applicable for small studies with few replicates (23). Transcripts per Million (TPM) was calculated by DESeq2. For significance testing, DESeq2 uses the Wald test to calculate the *P*-value. The Wald test calculates *P*-values from the subset of genes that pass an independent filtering step, and they are adjusted for multiple testing using the procedure of Benjamini and Hochberg (23). Expression with log_2_(fold-change) ≥ log_2_(2.0) and adjusted *P*-value <0.05 or log_2_(fold-change) ≤–log_2_(2.0) and adjusted *P*-value <0.05 was considered as differentially expressed (Dataset S6).

### Structural analysis of candidate TFs

Homology models of the candidate transcription factors YidZ, YfeC, YciT, YcjW, YdcN and YgbI were constructed using the SWISS-MODEL pipeline (24). Multiple templates were analyzed, and inference of the oligomeric state was based on the reported interface conservation scores to existing complexes of similar sequence identity. The structures were annotated using information in UniProt (25) and visualized with VMD (26).

## RESULTS

**Figure 1.**
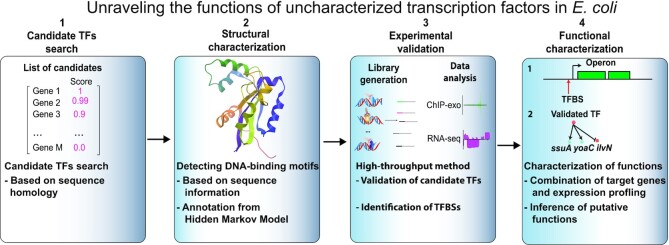
A systematic approach to identify and validate candidate transcription factors in *E. coli* K-12 MG1655. The approach used in this study can be divided into four steps: 1) we examined 40 computationally predicted candidate TFs from our previous study; 2) for each candidate TF, we highlighted its structural features based on the annotation from hidden Markov models; 3) we performed experimental validation using multiplexed ChIP-exo; and 4) we combined the binding sites with expression profiling data to characterize regulatory roles of representative TFs with a suite of experimental tests.

Here, we describe the discovery and characterization of candidate TFs in *E. coli* K-12 MG1655 following our previously reported and validated pipeline (10). First, we present an overview of the binding sites determined by multiplexed ChIP-exo for these candidate TFs, highlighting their structural and functional properties. We then describe the regulation of transcription initiation by these candidate TFs through a separate ChIP-exo screen for the RNAP holoenzyme. Next, we characterize the putative functions of 10 candidate TFs in *E. coli* to understand their biological roles (Figure 1). Finally, we provide further phenotypic analysis for the wild type and four mutant strains through deletion of either *yfeC*, *yciT*, *ybcM* or *ygbI*.

### Screening putative transcription factors in *E. coli* K-12 MG1655

Previously, we had generated a rank-ordered list of candidate TFs from a group of uncharacterized genes (‘y-genes’) using a homology-based algorithm (10). We experimentally tested 16 of the top hits from this list and verified that ten (62.5%) were indeed TFs. To expand this effort, in the present study, we selected an additional 40 y-genes from the list of candidate TFs and experimentally tested them by using multiplexed ChIP-exo (Table 1). Recently, several of the candidate TFs have been independently suggested to be TFs using *in vitro* assays: ComR (YcfQ) (27), YcjW (28), SutR (YdcN) (29), RcdB (YhjC) (30), NimR (YeaM) (31), CsqR (YihW) (32,33), YqhC (34,35). However, our results provide *in vivo* binding sites of these TFs, which is important for expanding the knowledge of the target genes for these TFs in *E. coli* K-12 MG1655.

**Table 1.** Overview of 40 candidate TFs with the predicted location of the helix-turn-helix (HTH) domain

Gene name	Locus_tag (b_number)	Total length (AA)	TF Family type*	Relative HTH position^#^
*yahB*	b0316	310	LysR	3–29%
*ybcM*	b0546	265	AraC	80–99%
*ybdO*	b0603	300	LysR	3–30%
*ybeF*	b0629	317	LysR	8–33%
*ybhD*	b0768	317	LysR	1.2–35%
*ycaN*	b0900	302	LysR	1–37%
*ycfQ*	b1111	210	TetR	5–39%
*yciT*	b1284	249	DeoR	1–24%
*ycjW*	b1320	332	GalR/LacI	0–17%
*ydcN*	b1434	178	N/A*	2–40%
*ydcR*	b1439	468	GntR	0–15%
*ydhB*	b1659	310	LysR	2–28%
*ydiP*	b1696	303	AraC	77–94%
*yeaM*	b1790	273	AraC	72–94%
*yebK*	b1853	289	N/A*	0–28%
*yedW*	b1969	223	CheY	0–56%
*yeeY*	b2015	309	LysR	2–28%
*yehT*	b2125	239	CheY	0–51%
*yfeC*	b2398	114	N/A*	0–50%
*yfeD*	b2399	130	N/A*	4–63%
*yfiE*	b2577	293	LysR	0–29%
*yfjR*	b2634	233	N/A*	N/A
*ygaV*	b2667	99	N/A*	11–99%
*ygbI*	b2735	255	DeoR	2–23%
*ygeR*	b2865	251	N/A*	N/A
*ygfI*	b2921	298	LysR	3–29%
*yggD*	b2929	169	N/A*	N/A
*yhjB*	b3520	200	LuxR	N/A
*yhjC*	b3521	299	LysR	1–28%
*yiaU*	b3585	324	LysR	2–35%
*yidL*	b3680	297	AraC	80–96%
*yidZ*	b3711	319	LysR	2–25%
*yihL*	b3872	236	GntR	1–31%
*yihW*	b3884	261	DeoR	3–31%
*yjhI*	b4299	262	IclR	3–29%
*yjjJ*	b4385	443	N/A*	N/A
*yneJ*	b1526	293	LysR	0–37%
*ynfL*	b1595	297	LysR	1–30%
*ypdC*	b2382	285	AraC	82–99%
*yqhC*	b3010	318	AraC	82–98%

Note, TF Family type* was annotated by the Hidden Markov Model (37).

N/A* indicates no annotation due to the lack of structural information.

Relative HTH position^#^ was calculated by the position of a HTH domain at the full length of protein sequence. N/A^#^ indicates the absence of a HTH domain in a given protein.

To predict the family types of candidate TFs, we employed Hidden Markov Models to annotate them based on the homology to the collection of known protein structures in the SUPERFAMILY 2 database (36) (Table 1, Dataset S7). We found that the majority of these 40 candidate TFs contain winged helix-turn-helix (HTH) DNA-binding domains, and can be grouped into different TF family types based on homology to known transcription factors (Supplementary Table S1) (37). These candidates can be classified into nine known TF family types (LysR, AraC, GntR, CheY, TetR, LuxR, GalR/LacI, IclR, DeoR) and one unknown group (due to the lack of structure information), which were listed in ‘TF family type’ (Supplementary Figure S2A). We then calculated the relative position of the HTH domain for all the candidate TFs, according to the start and end position of amino acids sequences (9) (Supplementary Figure S2B). Several candidate TFs (YfjR, YgeR, YggD, YhjB, YjjJ) do not have a predicted DNA-binding domain due to a lack of structural information, thus their relative HTH positions were annotated as N/A.

### Identifying the binding sites for candidate TFs

Next, to characterize binding sites of these candidate TFs on the genome, we constructed 40 myc-tagged strains corresponding to each candidate TF of interest and employed a multiplexed ChIP-exo method to increase the throughput of the assay (Supplementary Figure S3).

**Figure 2.**
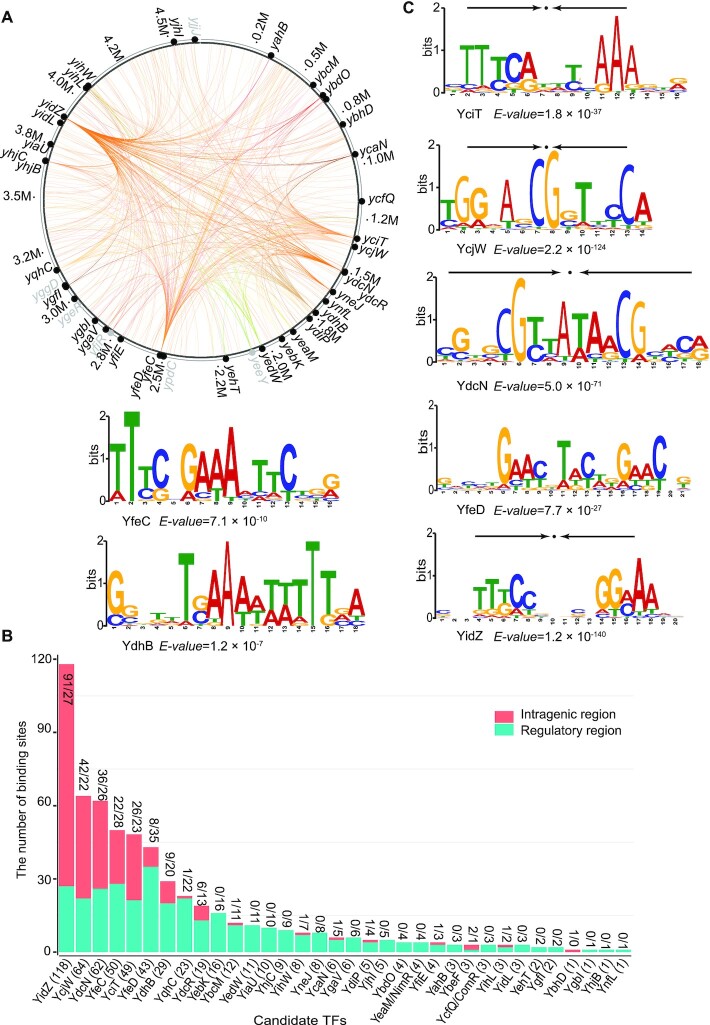
The global DNA binding profile for uncharacterized TFs. (**A**) Binding sites identified by a multiplexed ChIP-exo method are mapped onto the *E. coli* K-12 MG1655 genome to provide a network-level perspective of binding activity. Experimentally verified candidate TFs are shown in black, while TFs without binding peaks under tested conditions are shown in grey. The binding events for verified candidate TFs were labeled with colored lines. Each line indicates the interaction between a TF and its target genes. (**B**) 34 validated TFs have a varied number of binding sites between the intragenic region and the regulatory region. The numbers (#/#) above each bar indicate the number of sites that are located at the intragenic region and the regulatory region, respectively. The number (#) behind the name of a TF in the *x*-axis is the total number of binding sites for each validated TF. (**C**) The consensus sequence motifs for seven TFs determined by MEME. The height of the letters (in bits on the *y*-axis) represents the degree of conservation at a given position within the aligned sequence set, with perfect conservation being 2 bits. Arrows above motifs indicate the presence of palindromic sequences.

We obtained the binding profiles for all candidate TFs using the peak-calling algorithm MACE (16), and confirmed that 34 out of the 40 have DNA-binding affinities (Figure 2A). A total of 588 binding sites were identified for these candidate TFs (Figure 2B). Four of the six candidates, YgeR, YggD, YjjJ and YfjR, did not display any DNA binding, probably because they are non-HTH domain proteins (Table 1). It is likely that the remaining two proteins, YpdC and YeeY, are not activated under the test conditions in this study. They have therefore been excluded from further analyses.

For the 34 validated candidate TFs, we analyzed the conserved binding motifs using the MEME algorithm (38) and obtained consensus sequences for 7 TFs (Figure 2C). Specifically, we found that the consensus binding motifs for YciT, YcjW, YdcN and YidZ were palindromic. For some validated TFs (YbcM, YbdO, YcaN, YcfQ, YdiP, YedW, YihW and YqhC), although they had a limited number of binding sites, their binding sites exhibited sequence-specific patterns (Supplementary Figure S4).

The majority of promoters in *E. coli* are recognized by the sigma factor RpoD (σ^70^), also known as the housekeeping sigma factor (14). Thus we performed additional ChIP-exo experiments to investigate whether target genes of the 34 candidate TFs are also expressed by RNAP assembled with σ^70^ (Supplementary Figure S5A). We specifically focused on three combinations between RNA polymerase, RpoD, and candidate TFs: (i) RNAP + RpoD: a binding site is located upstream of a target gene, and both RNAP and RpoD recognize the promoter region of this gene; (ii) RNAP_only: a binding site is located upstream of a target gene, but only RNAP recognizes the promoter region (while RpoD could not recognize the promoter region, it is likely that alternative sigma factors could recognize this promoter region); and (iii) others: includes two scenarios; one where a binding site is located within the coding region, and the other where a binding site is located upstream of a target gene but neither RNAP or RpoD recognize the promoter region. Given these criteria, we identified 208 binding events belonging to type (i) and 75 binding events belonging to type (ii). Thus, a total of 283 binding events overlaps with RNAP for the 34 candidate TFs, accounting for 48% (283/588) of total binding sites (Supplementary Figure S5B).

### Deciphering regulatory roles of candidate transcription factors

Having verified whether candidate TFs were DNA-binding proteins, we next assessed their putative functions. We used the definition put forth by Shimada et al.—based on the number of target genes—to classify the regulatory nature of the TFs studied here (8). This definition uses four classes: (i) nucleoid-associated regulators (hundreds of target genes); (ii) global regulators (>100 target genes); (iii) local regulators (<100 target genes); and (iv) single-target regulators. In this study, 34 validated TFs were classified into the latter three types: 1 global regulator (type I), 29 local regulators (type II), and 4 single-target regulators (type III). In particular, we further inferred the putative biological roles of ten validated TFs (YidZ, YfeC, YciT, YdhB, YbcM, YneJ, YjhI, YfiE, YgbI and YnfL) based on annotated functions of their target genes (Table 2).

**Table 2.** The classification of 10 representative candidate TFs and proposed functions in *E. coli* K-12 MG1655

Gene^#^ (b-number)	Classification of candidate TFs (# of TFBSs)	Family Type	Binding sites associated with metabolic pathway	Proposed regulatory roles	Results
*yidZ* (b3711)	Type I (118)	LysR	Widespread, intragenic binding	Target genes have diverse functions	Figure 3
*yfeC* (b2398)	Type II (50)	N/A*	*chaAB, panD, grxC, pqqL*, *hybE*, *lpp, rpmH, rpmB*	*yfeC* mutant was reported to increase eDNA release (40)	Figure 4
*yciT* (b1284)	Type II (49)	DeoR	*ybiO*, *ybiV*, *ybiY*	A regulator involved in osmolarity	Figure 5
*ydhB^#^* (b1659)	Type II (29)	LysR	*ydhB*, *ydhC*	A regulator involved in purine metabolism	Supplementary Figure S6
*ybcM* (b0546)	Type II (12)	AraC	*ybcL*, *ucpA*	A regulator related to stress response	Figure 6
*yneJ^#^* (b1526)	Type II (8)	LysR	*sad*, *yneJ*	A regulator involved in glutamate metabolism	Supplementary Figure S7 (54)
*yjhI^#^* (b4299)	Type II (5)	IclR	*yjhG*, *yjhH*, *yjhI*	A regulator related to the energy conversion between pyruvate and glycolaldehyde	Supplementary Figure S8
*yfiE^#^* (b2577)	Type II (4)	LysR	*yfiE*, *eamB*	A regulator related to the control of a cysteine and O-acetylserine exporter	Supplementary Figure S9
*ygbI* (b2735)	Type III (1)	DeoR	*ygb*J, *ygbK*	A regulator involved in tartrate metabolism	Figure 7
*ynfL^#^* (b1595)	Type III (1)	LysR	*ynfL*, *ynfM*	A regulator involved in the control of arabinose efflux transporter	Supplementary Figure S10

*N/A indicates no prediction due to the lack of structural information.

Genes^#^ were analyzed and presented in the supplementary material.

For detailed analysis, 5 of 10 validated TFs in the three categories—one global regulator (YidZ), three local regulators (YfeC, YciT and YbcM), and one single-target regulator (YgbI)—were selected as representative TFs. To infer their regulatory roles, we combined the binding sites with gene expression profiling to analyze the most significant enrichment of pathways in which validated TFs are involved. The remaining five validated TFs can be found in the Supplementary Material.

#### A global regulator (type I), YidZ

**Figure 3.**
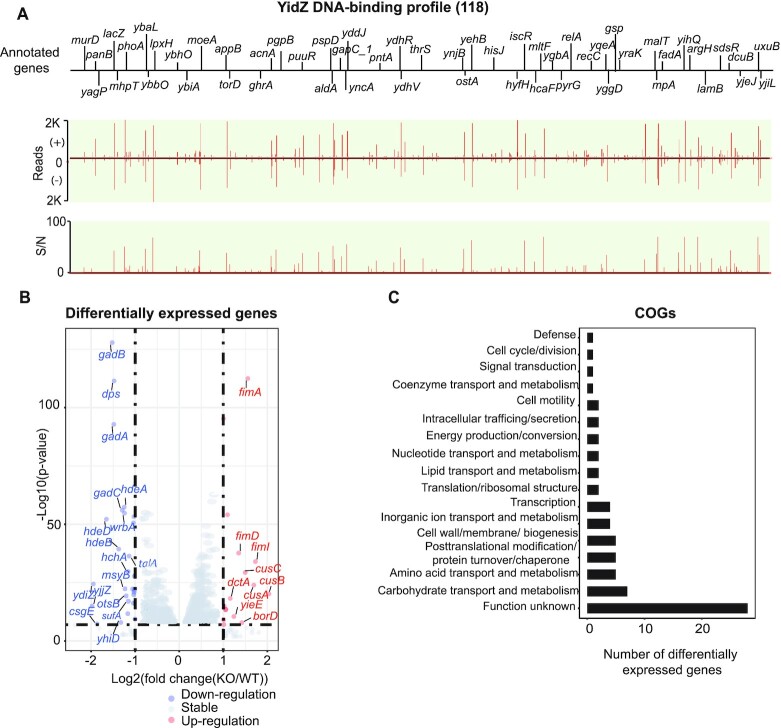
Using YidZ as an example to illustrate type I global regulators. (**A**) An overview of YidZ binding profile across *E. coli* K-12 MG1655 genome. 77% (91/118) of binding sites are located within the coding region while 23% (27/118) are located within the regulatory region. S/N denotes signal-to-noise ratio. (+) and (−) indicate reads mapped onto forward and reverse strands, respectively. (**B**) 74 genes were differentially expressed after deletion of *yidZ* (cut-off value is log_2_ fold-change ≥1, or ≤−1, and adjust *P*-value < 0.05). (**C**) Functional classification of genes regulated by YidZ. The functions of genes regulated by YidZ are diverse. Additionally, the biological significance of 38% (28/74) of genes is still unknown.

We identified 118 binding sites of YidZ (Figure 3A) and then enriched 108 out of the 118 binding sites at the high confidence (*E*-value = 1.2e–140, Figure 2C, Supplementary Figure S11). Based on SWISS-MODEL, YidZ was predicted to form the dimer or tetramer (Supplementary Table S2) (24).

To determine the relative location between YidZ binding *in vivo* and RNA polymerase, we integrated YidZ binding with the previous ChIP-exo data of RpoB and σ^70^. Among 27 YidZ intergenic bindings, we identified 12 binding sites at the promoters in the presence of core RNAP and σ^70^, 9 binding sites at the promoters in the presence of core RNAP, and 6 binding sites at the promoters in the absence of core RNAP and σ^70^ (Supplementary Figure S5B). Of the 91 intragenic binding sites, 34 are located inside the genes in the presence of core RNAP at the promoter DNA; the remaining 57 binding sites are in the absence of core RNAP at the promoters.

Finally, to explore the regulatory roles of YidZ, we compared the gene expression profile between the wild-type strain and the *yidZ* knockout strain using RNA-seq. With the deletion, we found that 19 of the 118 target genes were differentially expressed, indicating these genes are directly regulated by YidZ as a major regulator. Genes/operons associated with acid stress and amino acid transport and metabolism (*gadA*, *gadBC*, *hdeD*, *hdeAB-yhiD*) were down-regulated, while genes involved in carbohydrate transport and metabolism (*rbsD*, *malM*, *malE*, *malX*) were up-regulated (Figure 3B, Dataset S6). The remaining target genes from ChIP-exo were not differentially expressed after the deletion of *yidZ*.

Overall, we observed two notable features of the YidZ binding profile. First, YidZ has a large number of binding sites, with 77% (91/118) located within the coding regions and 23% (27/118) located within the intergenic regions. Second, YidZ is associated with diverse gene functions, based on Clusters of Orthologous Groups (COGs) annotations of differentially expressed genes (DEGs) (19) (Figure 3C). However, we did not find any significantly enriched COGs (*P* < 0.01), indicating that genes directly or indirectly regulated by YidZ are not, as a group, strongly associated with any specific function(s).

#### A local regulator (type II), YfeC

**Figure 4.**
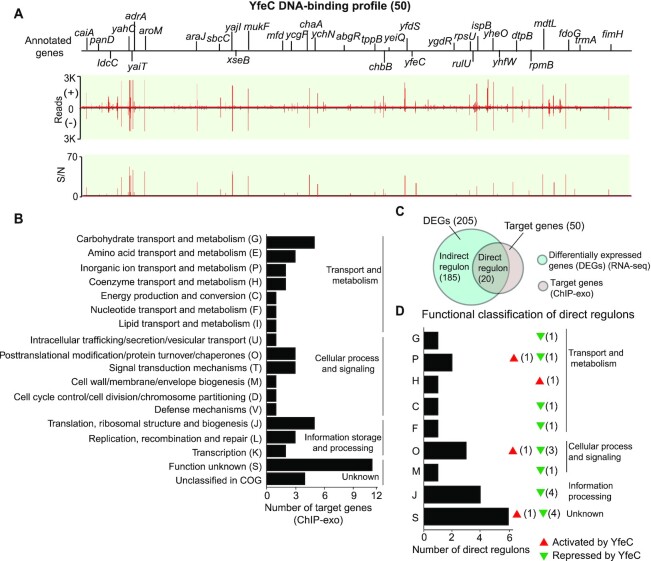
Using YfeC as an example to illustrate type II local regulators. (**A**) An overview of YfeC binding profile across *E. coli* K-12 MG1655 genome. 40% (20/50) of binding sites are located within the coding region while the remaining 60% (30/50) are located within the regulatory region. S/N denotes signal-to-noise ratio. (+) and (−) indicate reads mapped on forward and reverse strands, respectively. (**B**) Functional classification of target genes from YfeC binding sites. The enriched functions are in three groups: transport and metabolism, cellular process/signaling, and transcription/translation. (**C**) Comparison of ChIP-exo results and gene expression profiles to distinguish direct and indirect YfeC regulons under the test conditions. (**D**) Functional classification of genes directly regulated by YfeC. One-letter abbreviations for the functional categories are the same as those in panel B. Red triangles represent activation by YfeC. Green triangles represent repression by YfeC. The number behind the triangle represents the number of direct regulon genes.

We identified 50 YfeC binding sites in *E. coli* K-12 MG1655 (Figure 4A) and then enriched the sequence motif of YfeC (*E*-value = 7.1e−10, Figure 2C). The consensus DNA binding sequence showed that the TFBSs of YfeC enclose TTC-rich inverted repeats separated by 6-nt. It is likely that YfeC can form the homodimer in the cell as inferred from SWISS-MODEL (Supplementary Figure S12, Table S2).

Functional classification showed that 50 YfeC binding sites are involved in various functional groups, from DNA replication, transcription, translation, to cell envelope biogenesis (Figure 4B). To identify genes directly regulated by YfeC, we compared the gene expression profile between the wild-type strain and the *yfeC* knockout strain using RNA-seq, and found that 124 genes were up-regulated and 81 genes were down-regulated in the *yfeC* knockout strain, indicating that YfeC might be a dual regulator in *E. coli* K-12 MG1655 (Figure 4C, Supplementary Figure S13). Combining YfeC ChIP-exo results with the transcriptomic data, we found that 40% (20 of 50) of the genes with YfeC binding were differentially expressed, suggesting that these 20 genes are directly regulated by YfeC (Figure 4C, Supplementary Table S3). Of these 20 genes, 80% (16 of 20) are repressed by YfeC (Figure 4D). These data confirm that the regulation of YfeC is involved in various functional groups, such as nutrient transport and metabolism (*chaB*, *ychO*, *panD*), translation (*rpmH, rpmB*, *rpsU*), post-translational modification (*grxC*, *pqqL*, *hybE*), and cell envelope (*lpp*).

A previous study reported that single-gene deletion strains for genes *rna*, *hns*, *nlpI*, *rfaD* and *yfeC* altered eDNA production in *E. coli*. These mutations were related to general cellular processes, such as transcription (*rna*, *hns*), lipid transport (*nlpI*), cell envelope (*rfaD*), and unknown function (*yfeC*) (39). These results suggest that the *yfeC* gene is associated with the mutant phenotype-eDNA production in *E. coli*. Furthermore, although the underlying mechanisms remain unknown, the study hints that eDNA release might be related to multiple cellular processes rather than a single biological pathway. At this point there is no detailed molecular study to determine the mechanism of eDNA release regulated by YfeC in *E. coli*. Designing such a study may serve as the context for future work.

#### A local regulator (Type II), YciT

**Figure 5.**
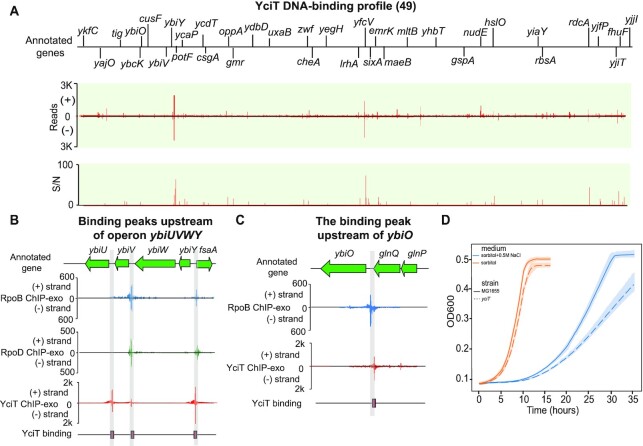
Using YciT as an example to illustrate type II local regulators. (**A**) An overview of YciT binding profile across *E. coli* K-12 MG1655 genome. S/N denotes signal-to-noise ratio. (+) and (−) indicate reads mapped on forward and reverse strands, respectively. (**B**) YciT binding peaks located upstream of operon *ybiUVWY* and gene *fsaA*. (**C**) The binding peak located upstream of gene *ybiO*. (**D**) Growth profiles of the wild type and *yciT* deletion strains in the absence and presence of 0.5 M NaCl in M9 minimal medium with 0.2% (w/v) sorbitol as the sole carbon source. Width of shaded bands represents standard deviation of the corresponding growth trajectory.

YciT was annotated as a DeoR-type putative transcription factor via the Hidden Markov Model. However, its *in vivo* DNA binding affinity had not been reported. Here, we identified 49 binding sites of YciT in *E. coli* K-12 MG1655 (Figure 5A), and then enriched the sequence motif of YciT binding sites (*E*-value = 1.8e−37, Figure 2C). To predict the putative functions of YciT, we assessed YciT binding sites and the functions of corresponding target genes. We found 47% (23 out of 49) of binding sites located within regulatory regions, indicating that these binding events may modulate target genes. Among these 23 binding sites, three target genes encode proteins involved in sugar metabolism, including sugar phosphatase (*ybiV*), a putative pyruvate formate-lyase activating enzyme (*ybiY*), and fructose-6-phosphate aldolase1 (*fsaA*) (Figure 5B). Some of the other genes encode products involved in membrane components, such as moderate conductance mechanosensitive channel YbiO (*ybiO*) (Figure 5C), copper/silver export system periplasmic binding protein (*cusF*), and outer membrane protein X (*ompX*). The remaining genes (such as *ykfC*, *ycaP*, *ydbD* and *yfdQ*) are of unknown function.

To confirm the regulation by YciT for these genes, we analyzed the transcriptomic data of the wild type and *yciT* deletion strain. It was found that target genes involved in metabolic pathways (*ybiV*, *ybiY*, *fsaA)* and membrane components (*cusF*) were indeed differentially expressed upon the deletion of the *yciT* gene (Supplementary Figure S14), indicating that YciT may participate in the control of the metabolic pathways and/or osmotic stress in *E. coli* K-12 MG1655.

To test these hypotheses, we evaluated the impact of *yciT* deletion on the growth of *E. coli* in M9 minimal media containing different carbon sources (glucose, fructose, sorbitol), and found that the deletion of the *yciT* gene did not reveal significant growth deficiencies compared to the wild type strain. However, the final OD_600_ of the *yciT* deletion strain at the stationary phase was slightly lower than the wild type strain (Supplementary Figure S15). Since the physiological roles of enzymes (YbiV, YbiY, and FsaA) regulated by YciT are not yet fully understood, little is known about the impact of YciT on the metabolic pathways.

Furthermore, we assessed the effects of osmotic stress on *E. coli* grown in M9 minimal medium with sorbitol as the sole carbon source (Figure 5D). We found osmotic stress induced growth retardation in the wild type and *yciT* deletion strains. Specifically, high osmolarity resulted in impaired growth and slowed the growth rate of the *yciT* deletion strain. Thus, we demonstrated that YciT is involved in the control of osmolarity in *E. coli* K-12 MG1655.

#### A local regulator (type II), YbcM

**Figure 6.**
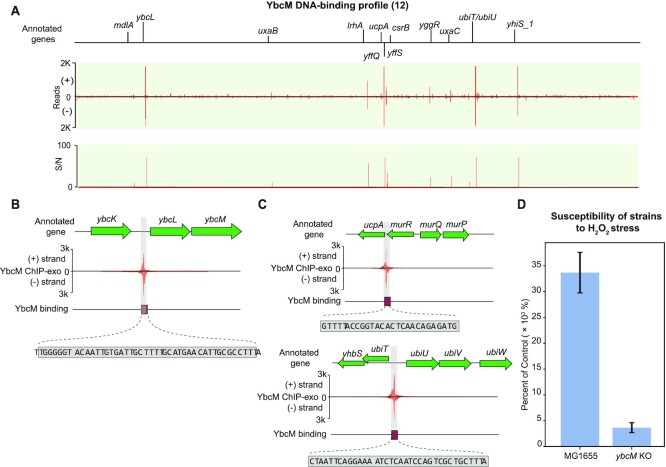
Using YbcM as an example to illustrate type II local regulators. (**A**) An overview of YbcM binding profile across *E. coli* K-12 MG1655 genome. S/N denotes signal-to-noise ratio. (+) and (−) indicate reads mapped on forward and reverse strands, respectively. (**B**) In-depth mapping of the YbcM binding site explains how YbcM interacts with the upstream region of operon *ybcLM*. The rectangle denotes the sequence recognized by YbcM. (**C**) A zoom-in of YbcM binding peaks upstream of genes *ucpA* and *ubiT*. (**D**) Susceptibility of the wild type and *ybcM* deletion strains under oxidative stress. Both the wild type and *ybcM* deletion strains (mid-log phase cells) were treated with 60 mM H_2_O_2_ for 15 min. The sensitivity of cells to the lethal effects was expressed as percent survival of treated cells relative to that of untreated cells determined at the time of treatment. The survival rate of the wild type strain was 8-fold higher than that of the *ybcM* deletion strain.

The *ybcM* gene was found by screening genes whose products protect *E. coli* from lethal effects of stresses (40). But there are no *in vivo* assays to confirm its DNA binding affinity. To determine the binding sites, the ChIP-exo experiment for YbcM was conducted under oxidative stress. We identified 12 binding sites in *E. coli* K-12 MG1655 (Figure 6A). 92% (11/12) of the binding sites are located upstream of target genes. We found one binding site located upstream of operon *ybcLM*, indicating its autoregulation (Figure 6B). The gene *ybcL* encodes the periplasmic protein YbcL, and has sequence and structural similarity to rat/human RKIP (Raf kinase inhibitor protein), which modulates signal transduction pathways (41).

To predict the functions of YbcM, we examined 12 binding sites and their functions, and found that there are two important binding sites involved in stress response. The first was located upstream of the gene *ucpA*, encoding the oxidoreductase UcpA (Figure 6C, upper panel). Overexpression of *ucpA* in plasmids was previously shown to lead to improved tolerance to furan (42), a chemical likely generating oxidative stress. The other divergent binding site was located between operons *ubiT-yhbS* and *ubiUV* (Figure 6C, bottom panel). Here, the *ubiT* gene encodes anaerobic ubiquinone biosynthesis accessory factor UbiT, *yhbS* encodes putative N-acetyltransferase YhbS, and *ubiUV* encodes ubiquinone biosynthesis complex UbiUV. Another gene, *ubiW*, near the operon *ubiUV*, encodes putative luciferase-like monooxygenase. We also identified a consensus YbcM binding motif in the regulatory region of these target genes (Supplementary Figure S4). Taken together, this data suggests that YbcM is a regulator responsible for the oxidative stress response in *E. coli* K-12 MG1655.

To confirm YbcM’s physiological role, the survival rate of the wild type and *ybcM* deletion strains were compared under oxidative stress conditions (Figure 6D). The survival rate of the wild type strain was 8-fold higher than the *ybcM* deletion strain after 15 min 60 mM H_2_O_2_ treatment. This observation confirms the involvement of YbcM in the reactive oxygen species (ROS) stress response.

#### A single-target regulator (type III), YgbI

**Figure 7.**
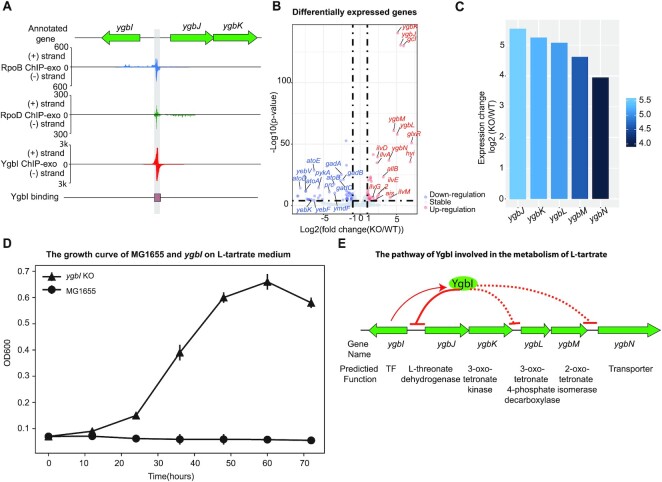
Using YgbI as an example to illustrate type III single-target regulators. (**A**) A zoom-in mapping of YgbI, RpoB, and RpoD binding events explains how YgbI binds onto the upstream region of operon *ygbJK*, covering the region that RpoB can recognize. Thus, YgbI blocks the transcription initiation of operon *ygbJK* when YgbI is active. (**B**) 113 genes were differentially expressed after deletion of *ygbI* (cut-off value is log_2_ fold-change ≥1, or ≤−1, and adjust *P*-value < 0.05). (**C**) Expression changes for genes in the *ygbI* deletion strain in a set of genes (*ygbJKLMN*) near the binding peak, compared to the wild type strain. (**D**) Growth of *E. coli* K-12 MG1655 and *ygbI* deletion strains on 20 mM l-tartrate medium, dicarboxylic acid. Circle markers represent growth of the wild type strain. Triangle markers represent growth of the *ygbI* deletion strain. (**E**) The proposed mechanism for the regulatory role of YgbI. When YgbI is present (active), it directly represses the promoter of the operon *ygbJK*, and indirectly inhibits the expression of operon *ygbLM* and gene *ygbN*. When gene *ybgI* is knocked out, it leads to de-repression of the operons *ygbJK*, *ygbLM* and *ygbN*.

In this study, we identified a single divergent binding site between the *ygbI* and *ygbJ* genes, indicating the autoregulation of *ygbI* (Figure 7A). We also found that this binding site overlaps the promoter region of the gene *ygbJ*. This observation strongly suggested that the overlap competes with the RNAP binding site, repressing the expression of downstream genes (*ygbJ*, *ygbK*).

To examine this assumption about the regulation of YgbI, we compared gene expression profiling between the wild type and the *ygbI* mutant (Figure 7B). The results showed that the expression of a cluster of genes (*ygbJ*, *ygbK*, *ygbL*, *ygbM*, *ybgN*) are upregulated after the deletion of *ygbI* (Figure 7C). This suggests that YgbI regulates the downstream gene cluster (*ygbJKLMN*) as a repressor, which is consistent with the prediction of a regulatory effect.

Previous studies reported that the downstream gene cluster *ygbJKLM* had putative functions in catabolic pathways for acid sugars (43), and hypothesized that the *E. coli* K-12 strain carrying mutations in the *ygbI* gene would provide a growth benefit on the tartrate medium (44). To verify the function of YgbI, the growth profiles of the wild type and the *ygbI* deletion strain were measured in 20 mM l-tartrate medium. Although the wild type strain does not grow on l-tartrate medium, the *ygbI* deletion strain could grow on l-tartrate (Figure 7D). Taking these factors into consideration, the potential pathway that YgbI is involved in was proposed as follows: when YgbI is present and active *in vivo*, it directly binds to the promoter of the operon *ygbJK*, and indirectly inhibits the expression of the genes *ygbLM* and *ygbN*. When the gene *ybgI* is knocked out, it leads to de-repression of operons *ygbJK* and *ygbLM* and the gene *ygbN* (Figure 7E). Based on the putative function of genes (*ygbJKLMN*), we suggest that YgbI is a repressor involved in the catabolic pathway for l-tartrate in *E. coli* K-12 MG1655.

## DISCUSSION

Despite extensive research over many decades focused on the *E. coli* genome, around 35% of its genes are still poorly characterized, including some uncharacterized transcription factors (10,45). Our primary goal in this study was to generate a large data set to further identify DNA-binding proteins from a pool of uncharacterized proteins in *E. coli* K-12 MG1655. We used a systematic approach to validate 34 computationally predicted transcription factors and employed a multiplexed ChIP-exo method to characterize binding sites and classify this experimental evidence for each TF. Next, we compared the binding profiles of the candidate TFs with binding peaks for RNAP holoenzyme, which generated a total of 283 (out of 588 sites) that are likely to regulate a nearby promoter (Dataset S4), and provide a coarse-grained functional prediction. Finally, we inferred the putative functions for ten of these candidate TFs (YidZ, YfeC, YciT, YdhB, YbcM, YneJ, YjhI, YfiE, YgbI, YnfL), and verified the biological roles of the representative TFs with detailed analysis. The implications of our results are below.

First, our study collected a large dataset of 588 TFBSs and expanded the total number of verified TFs in *E. coli* K-12 MG1655, close to the estimated total number of 280 (Supplementary Figure S16). Comparative analysis of binding sites of the TFs and RNAP enables the identification of target genes that are recognized by RNA polymerase complexes. The 283 RNAP binding sites among a total of 588 TFBSs means that almost half of the binding sites are likely to regulate a nearby promoter under the test conditions. Also, the interaction between RNAP and the recognition sequence at the promoter region may change depending upon the test conditions. It is possible that some TFBSs that are not identified by RNAP may be recognized by the RNAP complex under different conditions. Furthermore, discovering all of the TFs is fundamental to fully understanding the key role TRNs play in enabling bacteria to modulate the expression of thousands of genes in response to environmental and genetic perturbations (46). This study has brought us closer to revealing the identity of all the TFs in *E. coli* K-12 MG1655.

Second, we used the definition of TFs reported by Shimada et al., to classify candidate TFs into three groups: type I regulators, type II regulators, and type III single-target regulators (8). This classification was based on the number of genes bound by TFs as determined from the systematic evolution of ligands with exponential enrichment (SELEX) (47). Our rationale for using this classification was twofold: (i) the multiplexed ChIP-exo method employed here offers a similar readout to SELEX (i.e., the number of target genes), allowing for its application in the same context; and (ii) it has a successful track record of assigning annotations (e.g. ‘global’ or ‘local’ regulator) prior to a full understanding of the functions of the validated TFs, helping to guide their future study. Thus, we employed this classification based on the number of target genes shown by genome-wide experiments. We expect that a detailed characterization of these validated TFs will help us develop a comprehensive understanding of transcriptional regulation in *E. coli* K-12 MG1655.

Third, we did not identify binding sites for six of the candidate TFs tested in this study (YgeR, YggD, YjjJ, YfjR, YeeY, YpdC). There may be two reasons for this. The first is the false-positive predictions of candidate TFs due to the limitations of the sequence homology search. Specifically, YgeR has been recently re-annotated as putative lipoprotein involved in septation (48). YggD has been verified as fumarase E (49). Overexpression of YjjJ increases toxic effects in *E. coli*, thus *yjjJ* is likely to be a toxin (50). YfjR is predicted as a putative TF involved in biofilm formation (51), but a recent study that searched for novel TFs involved in biofilm formation has not validated this prediction (30). A second reason for failed prediction is that we may need to test for DNA-binding activity under the active conditions. YeeY and YpdC are annotated as a LysR-type regulator with a C-terminal HTH domain and an AraC-type regulator with a C-terminal HTH domain, respectively (Table 1). Thus they may have regulatory functions under the appropriate growth conditions.

Fourth, while we identified additional TFs with the experimental data, we did not fully decipher mutant phenotypes. For example, we identified YciT as a TF and found that it directly regulated multiple target genes (*fsaA*, *ybiY*, *ybiV*). This result hinted at an uncharacterized pathway composed of genes encoding DUF1479 domain-containing protein (*ybiU*), a sugar phosphatase (*ybiV*), a putative pyruvate formate lyase (PFL) (*ybiW*), a putative pyruvate formate-lyase activating enzyme (PFL-AE) (*ybiY*), and a fructose-6-phosphate aldolase1 (FSA) (*fsaA*) (Supplementary Figure S17). However, these enzymes and their corresponding substrates are rare and have not been identified. Little is known about their physiological roles in *E. coli* K-12 MG1655 (52). These bottlenecks may pose challenges in fully examining mutant phenotypes. Studying these enzymes should provide insight into the biological roles of YciT.

Finally, a collection of TFBSs data sets will lay the foundation for understanding the mechanisms of transcriptional regulation. In this study, we discovered that YfeC regulates multiple cellular processes in *E. coli* K-12 MG1655. Previous studies had not delved into a possible relationship between eDNA release and YfeC. Therefore, we employed a *yfeC* mutant to better understand any possible connections. The common mechanism of eDNA release in bacteria is through membrane vesicles (MVs) secretion (39). Thus eDNA production relies on several biological processes: (i) DNA replication, to produce DNA for secretion (referred to as eDNA); (ii) nutrient transport and metabolism, to generate lipid metabolism for MVs; (iii) energy conversion, to produce energy for the conversion of metabolism and the secretion of MVs; (iv) transcription and translation, to produce the proteins for the assembly of MVs; (v) post-translational modification, protein turnover, and chaperones, to modify and fold the proteins for secretion and (vi) cell wall/envelope biogenesis, to repair the cell wall after the secretion of eDNA (Supplementary Figure S18) (53). As a repressor, YfeC participates in many cellular processes, including lipid metabolism, translation, post-translational modification, and cell wall/envelope biogenesis. Accordingly, these corresponding biological processes are up-regulated after the deletion of *yfeC*. We proposed that the deletion of the *yfeC* gene may hasten these cellular processes, leading to eDNA release. Taken together, this study significantly expands the size of the TFs with experimental evidence, broadening our knowledge of transcriptional regulation in *E. coli* K-12 MG1655.

## DATA AVAILABILITY

The whole dataset of ChIP-exo and RNA-seq has been deposited to GEO with the accession number of GSE159777 and GSE159658, respectively.

## Supplementary Material

gkab735_Supplemental_Files
