# Supplementary material for: Unraveling the functions of uncharacterized transcription factors in Escherichia coli using ChIP-exo
Source: Nucleic Acids Res. 2021 Aug 24;49(17):9696–710. doi: 10.1093/nar/gkab735 (PMC8464067; doi:10.1093/nar/gkab735)
Supplement: gkab735_Supplemental_Files [file gkab735_supplemental_files.zip › Supplementary materials_revision.pdf]

Unraveling the functions of uncharacterized transcription factors in *Escherichia coli* using ChIP-exo

Ye Gao[1,2], Hyun Gyu Lim[2], Hans Verkler[2], Richard Szubin[2], Daniel Quach[2], Irina Rodionova[2], Ke Chen[2], James T. Yurkovich[2], Byung-Kwan Cho[3], Bernhard O. Palsson [2,4,5,6]

Dataset S1 The strains used in this study

Dataset S2 The conditions for genome-wide experiments

Dataset S3 The statistics for ChIP-exo experiments

Dataset S4 The binding sites of candidate TFs in *E. coli* K-12 MG1655

Dataset S5 The dataset of gene expression (TPM)

Dataset S6 The differentially expressed genes of representative candidate TFs in *E. coli* K-12 MG1655

Dataset S7 The homology analysis of candidate TFs using a library of Hidden Markov models

Supplementary material

Deciphering regulatory targets of candidate transcription factors

Local regulator (type II), YdhB. We identified 29 binding sites of YdhB in *E. coli* K-12 MG1655. There is one divergent binding peak among them located between genes *ydhB* and *ydhC* (**Supplementary Figure 6**). The gene *ydhB* encodes the putative LysR-type DNA-binding transcriptional regulator. The gene *ydhC* encodes the putative transporter YdhC which has been implicated in arabinose efflux (1). It is likely that YdhB represses transcription from the ydhC promoter and also interferes with its own transcription.

Local regulator (type II), YneJ. We identified 8 binding sites of YneJ in *E. coli* K-12 MG1655 (**Supplementary Figure 7**). We found that YneJ directly controls the gene *sad* encoding succinate-semialdehyde dehydrogenase Sad. YneJ binds to the promoter of the operon *sad-yneH-yneG*, repressing gene expression. However, the expression of the operon *sad-yneH-yneG* is activated during putrescine utilization (2). Thus, these results implicate that YneJ is involved in putrescine utilization.

Local regulator (type II), YjhI. We identified 5 binding sites of YjhI in *E. coli* K-12 MG1655. We observed that YjhI has a binding site located upstream of operon *yjhIHG* (**Supplementary Figure 8**). The gene *yjhI* encodes the putative DNA-binding transcriptional regulator YjhI, *yjhH* encodes putative 2-dehydro-3-deoxy-D-pentonate aldolase, and *yjhG* encodes D-xylonate dehydratase. This operon regulates energy

conversion between pyruvate and glycolaldehyde. Thus, we inferred that YjhI is likely associated with the conversion between pyruvate and glycolaldehyde.

Local regulator (type II), YfiE. We identified 4 binding sites of YfiE in *E. coli* K-12 MG1655, and found that YfiE has one divergent binding peak between genes *yfiE* and *eamB* (**Supplementary Figure 9**). The gene *yfiE* encodes the putative LysR-type DNA-binding transcriptional regulator YfiE, and the gene *eamB* encodes the cysteine/O-acetylserine exporter EamB. It is likely that YfiE is in the control of a cysteine and O-acetylserine exporter.

Singly-target regulator (type III), YnfL*.* We identified a single target gene for YnfL in *E. coli* K-12 MG1655. This single binding site was located between the genes *ynfL* and *ynfM* (**Supplementary Figure 10**). The gene *ynfL* encodes the putative DNA-binding regulator, and the gene *ynfM* encodes the putative transporter YnfM. A previous study reported that YnfM is implicated in arabinose efflux (1). Therefore, it is possible that YnfL is a single-target regulator that controls the transporter YnfM that is involved in arabinose efflux.

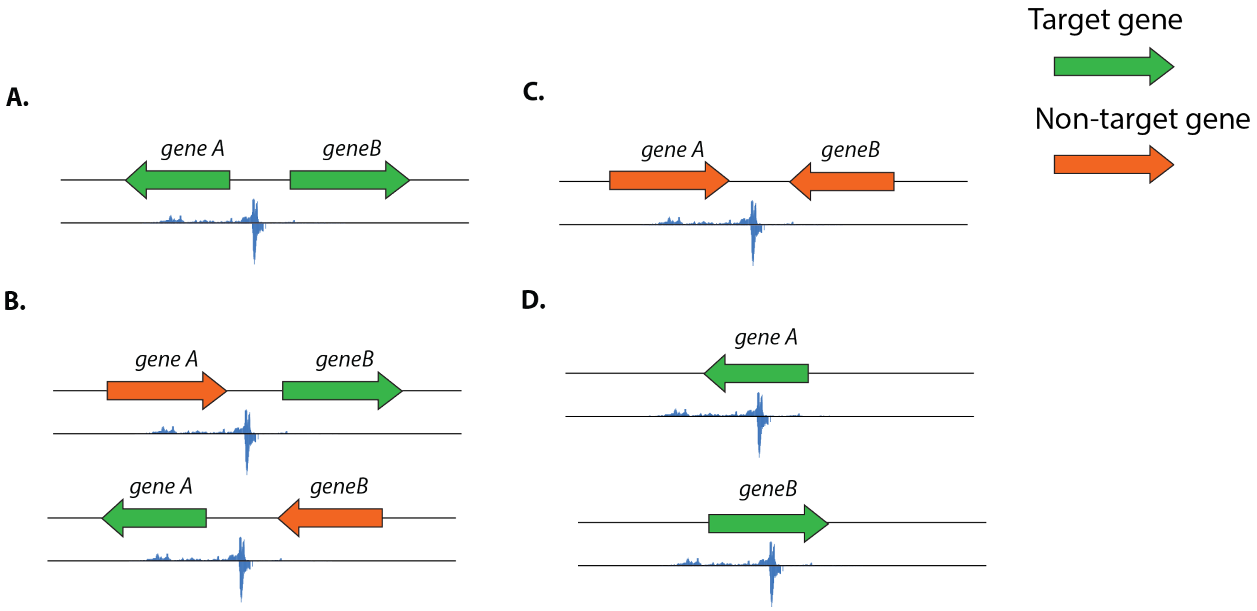

Supplementary Figure 1 The identification of target genes in peaking calling steps.

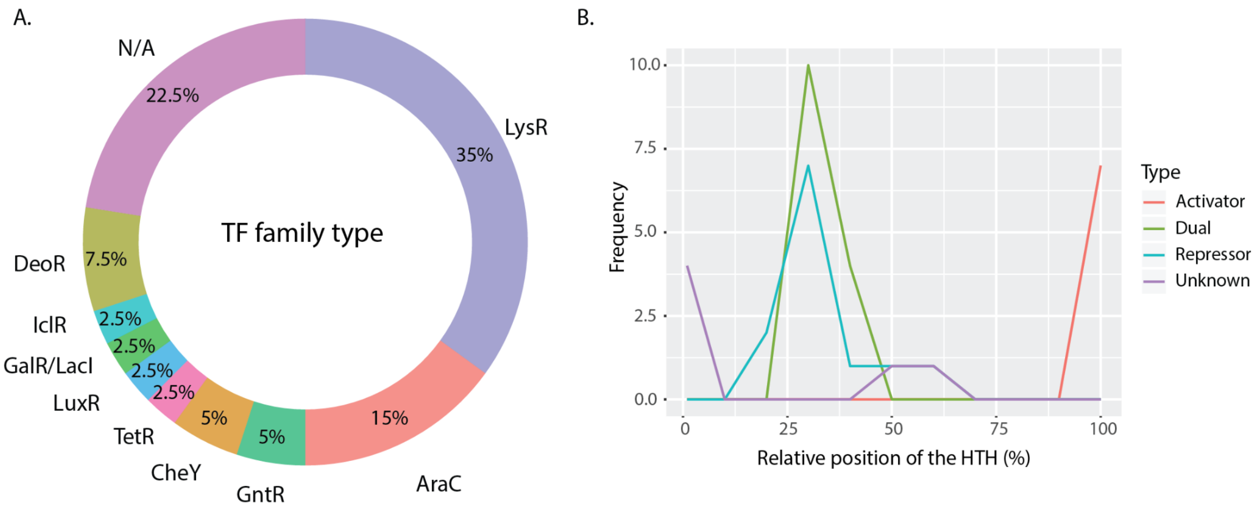

Supplementary Figure 2 The predicted family type for the 40 candidate TFs.
(A) The classification of transcription factor family types for 40 candidate TFs. (B) Helix-Turn-Helix (HTH) distribution of 40 candidate TFs analyzed in this study. The distribution of the relative HTH location is shown for different subsets of the collection. On the x-axis, 0% represents the N-terminus and 100% represents the C-terminus of the protein. The y-axis is the number of candidate TFs. The four types of predicted results were colored in the legend. “Unknown” indicates that there is no prediction due to the lack of the information about HTH domain in the protein.

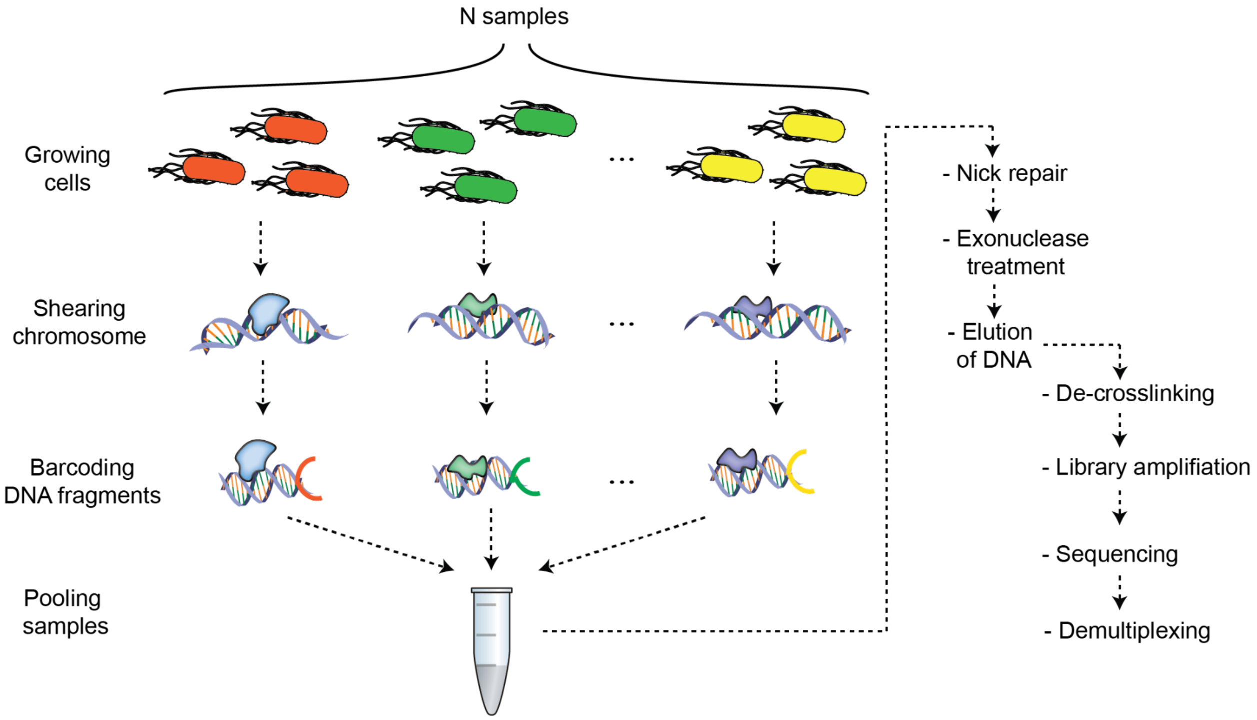

Supplementary Figure 3 The workflow of a multiplexed ChIP-exo system.
Cell cultures are crosslinked using formaldehyde. The complex (DNA bound to each candidate TF) is then extracted and fragmented using a sonicator. Sheared DNA fragments are immuno-precipitated with an antibody against the myc-epitope. After ligation of the first adapter with specific indices, N fragmented samples have distinct indices for each other. They can then be pooled and processed together (on-bead enzymatic reactions of the ChIP-exo method). The whole process can be repeated M times to generate N*M libraries. DNA recovered from the ChIP material can be directly amplified by PCR and deep-sequencing by Illumina next-generation sequencing technology.

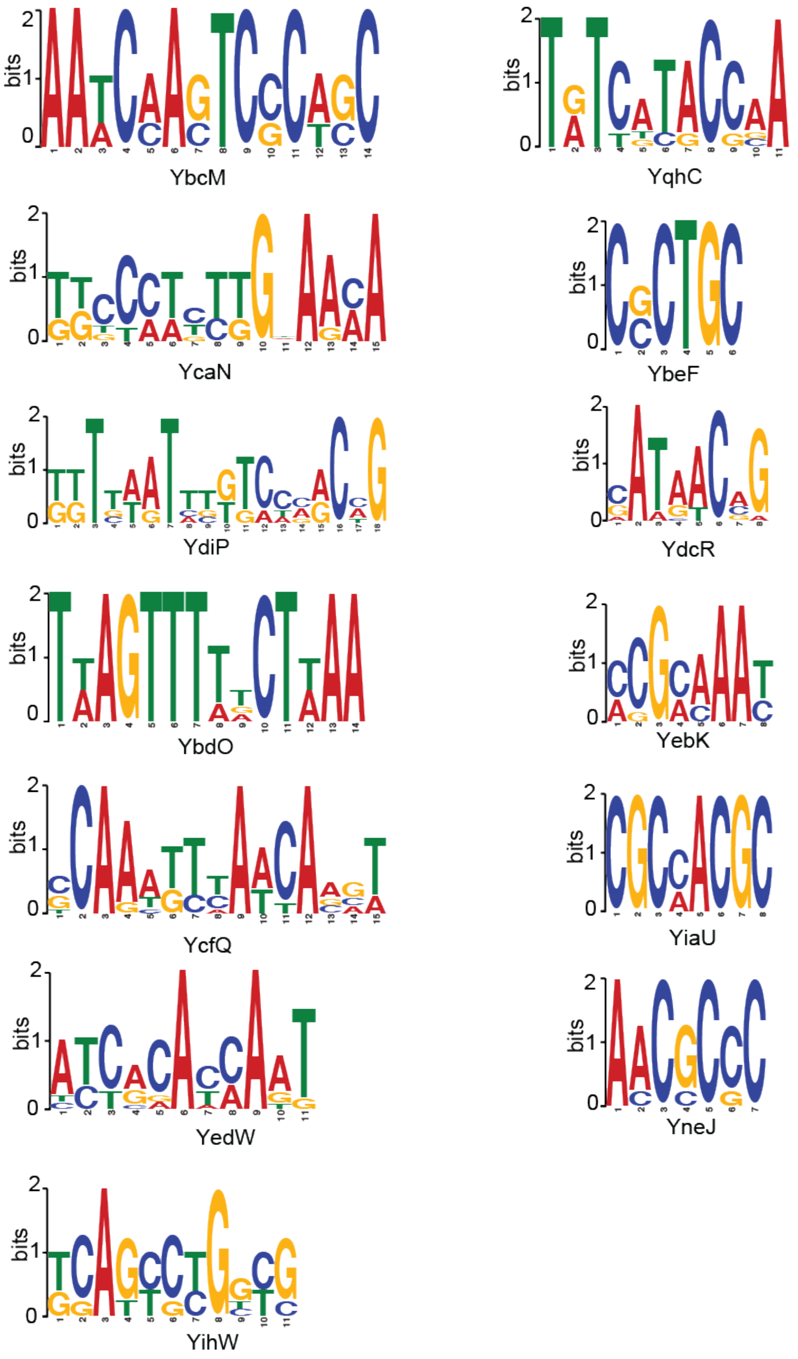

Supplementary Figure 4 The sequence motifs for candidate TFs.
The height of the letters (in bits on the *y*-axis) represents the degree of conservation at a given position within the aligned sequence set, with perfect conservation being 2 bits.

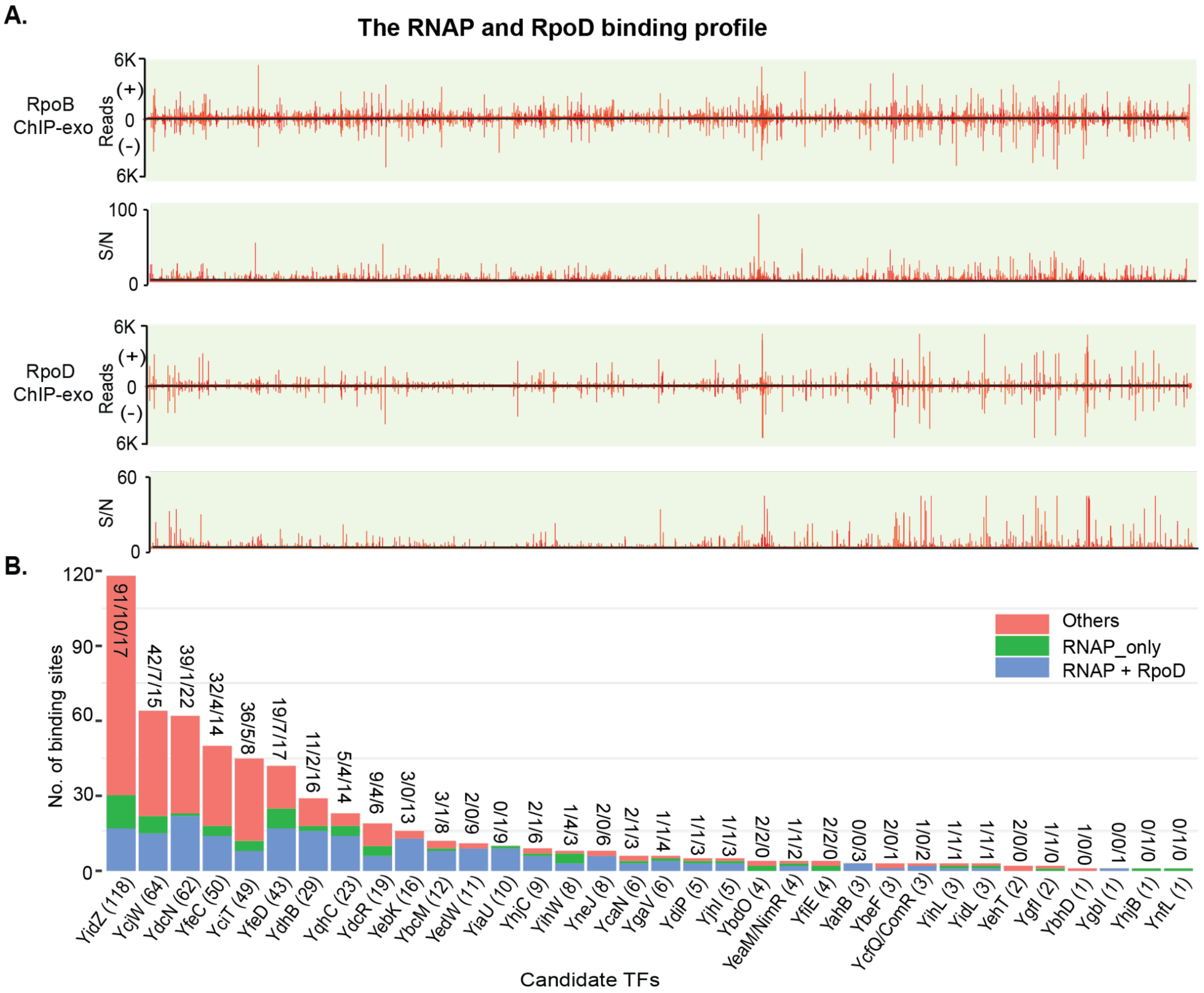

Supplementary Figure 5 Mapping binding sites with RNA polymerase activities.
(A) The RNAP and RpoD binding sites in the genome of *E. coli* K-12 MG1655. Upper panel: overview of RpoB binding sites. Bottom panel: overview of RpoD binding sites. S/N denotes signal-to-noise ratio. (+) and (−) indicate reads mapped on forward and reverse strands, respectively. (B) The classification of the combinations between RpoB, RpoD, and binding sites for 34 candidate TFs. The numbers (#/#/#) above each bar indicates the number of sites that fit for different types of combinations (in the order of "Others, RNAP_only, and RNAP + RpoD"). Others include two scenarios: one where a binding site is located within the coding region, the other where a binding site is located upstream of the coding region but neither RNAP or RpoD recognize the promoter region. The number (#) behind the TF name in the *x*-axis is the total number of binding sites for each validated TF.

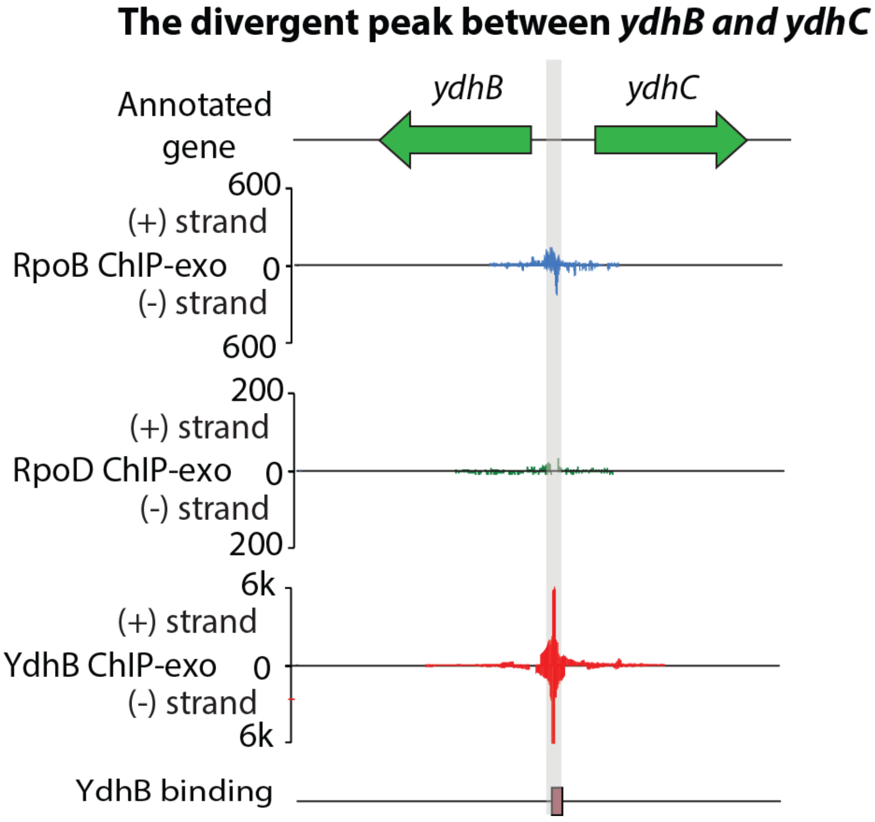

Supplementary Figure 6 A zoom-in of the divergent peak between *ydhB* and *ydhC*.

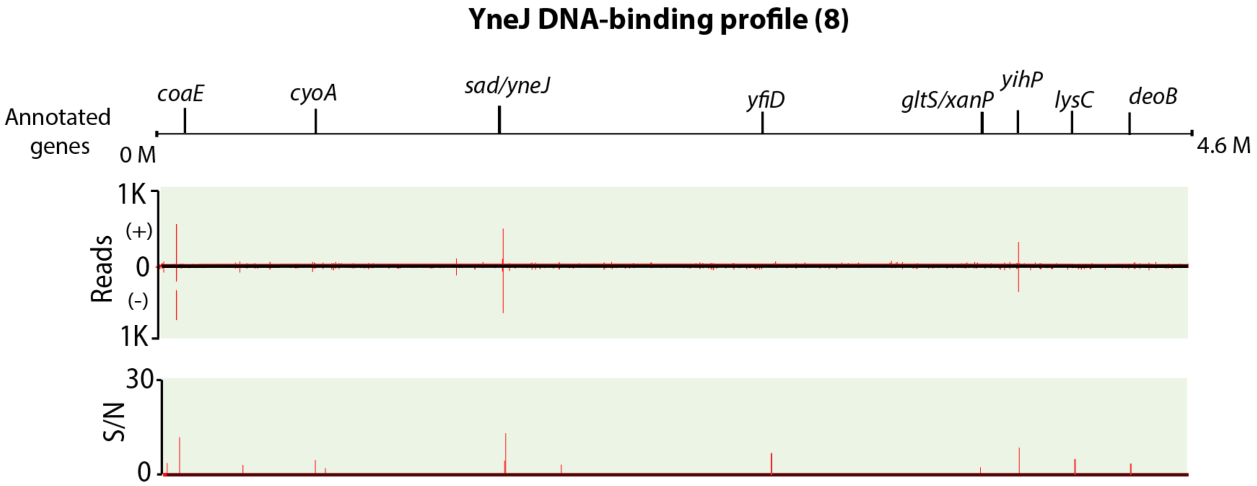

Supplementary Figure 7 An overview of YneJ binding profile across *E. coli* genome.

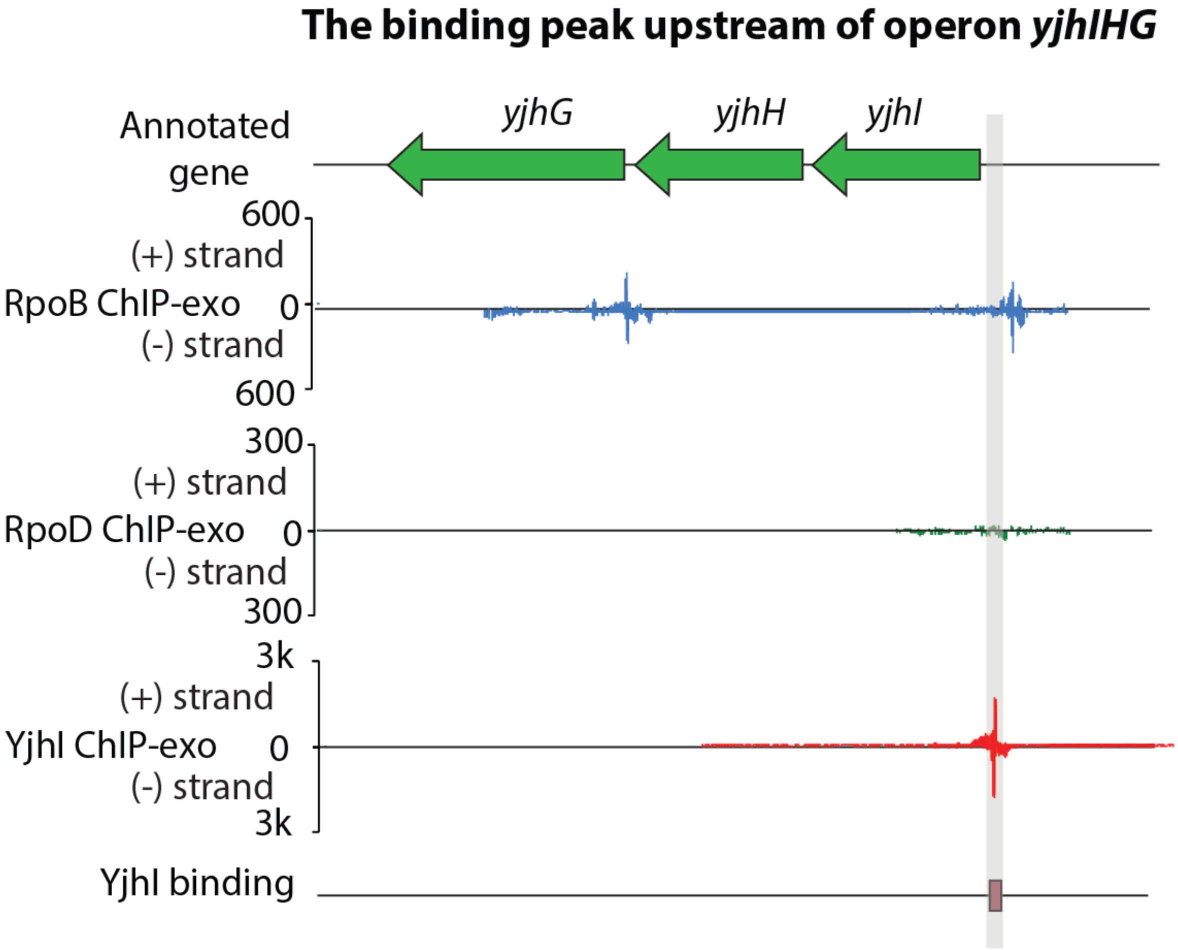

Supplementary Figure 8 A zoom-in of the binding peak upstream of operon *yjhIHG*.

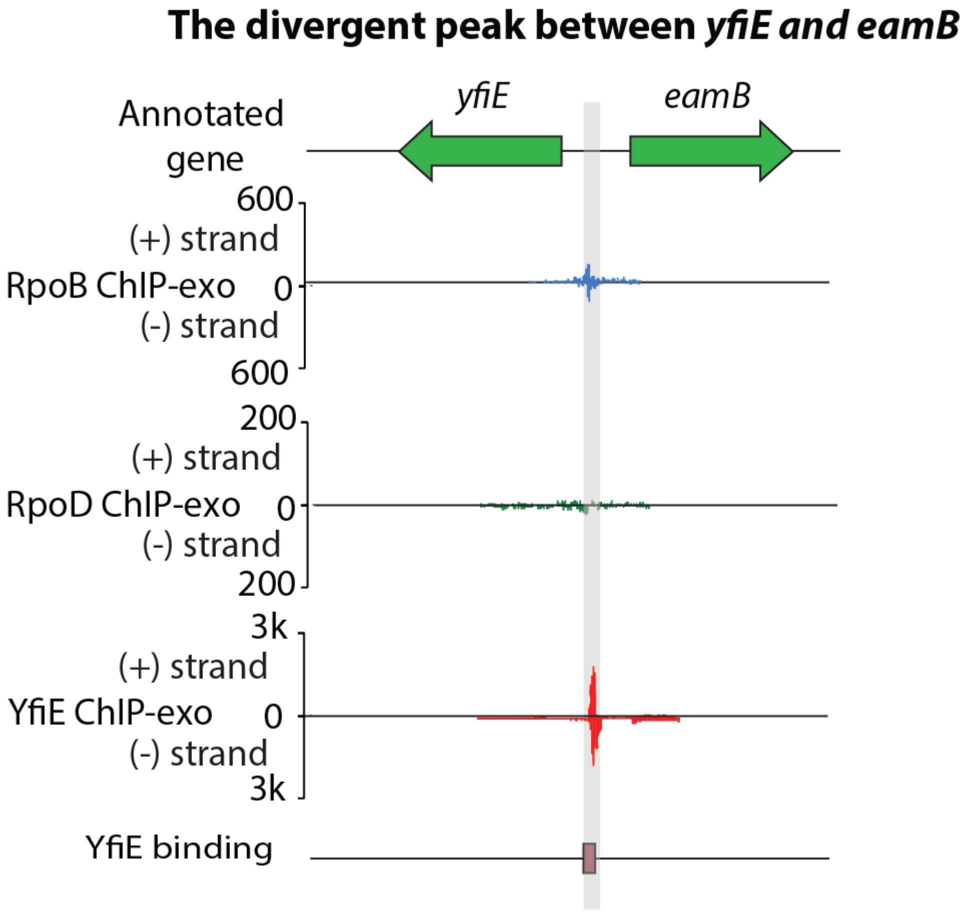

Supplementary Figure 9 A zoom-in of the divergent peak between *yfiE* and *eamB*.

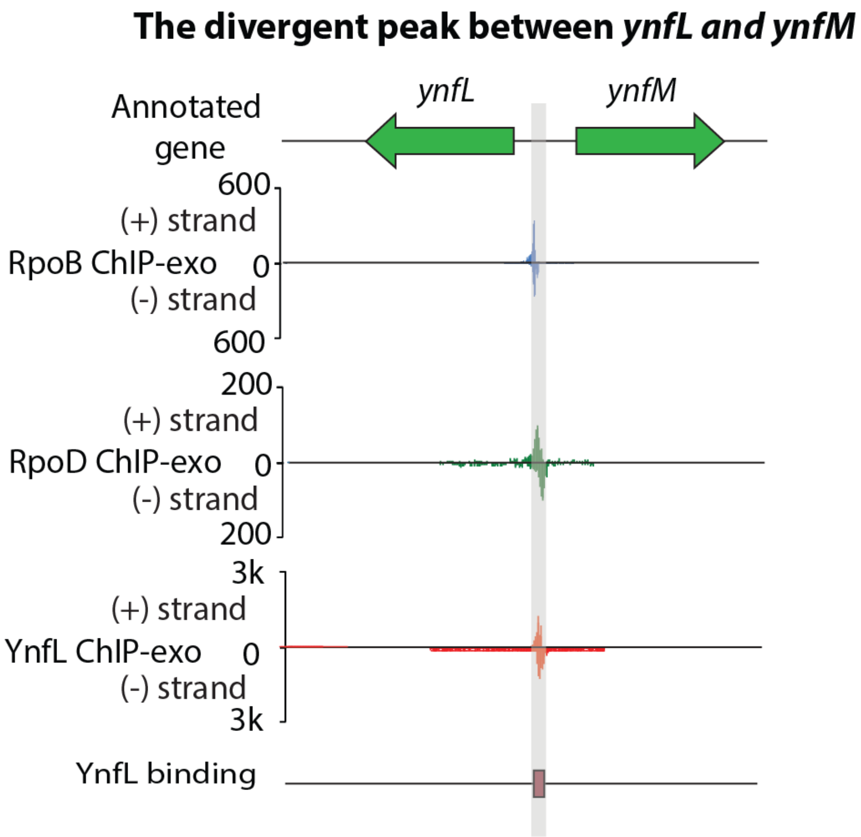

Supplementary Figure 10 A zoom-in of the divergent peak between *ynfL* and *ynfM*.

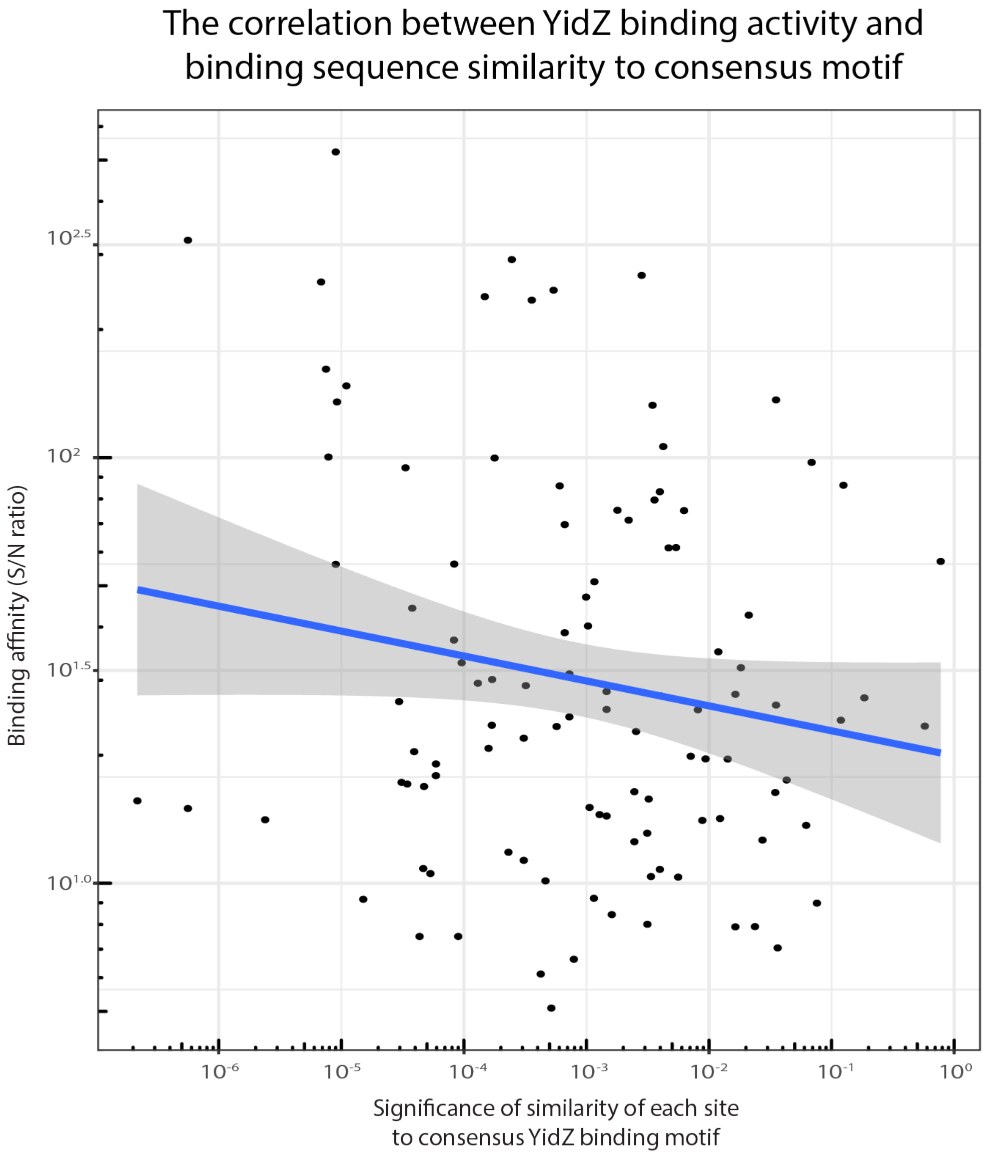

Supplementary Figure 11 Relationship between YidZ binding affinity and binding sequence similarity to consensus motif.

The signal-to-noise (S/N) ratio of each YidZ-binding peak was assigned to the *in vivo* relative binding affinity of each site. The significance of similarity of each site to the consensus YidZ binding motif was represented by statistical significance (*p*-value) computed from the match score of the site with the position specific scoring matrix for the motif. More differences between the binding sequence and consensus motif resulted in increasingly lower binding affinity.

Predicted YfeC structure

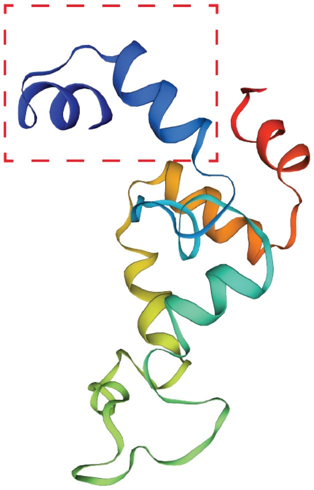

Supplementary Figure 12 Predicted 3D structure of YfeC (monomer with the range of AA 2-111).

The N-terminal region, labelled by a red dashed rectangle, is predicted as a DNA-binding domain by Hidden Markov Models.

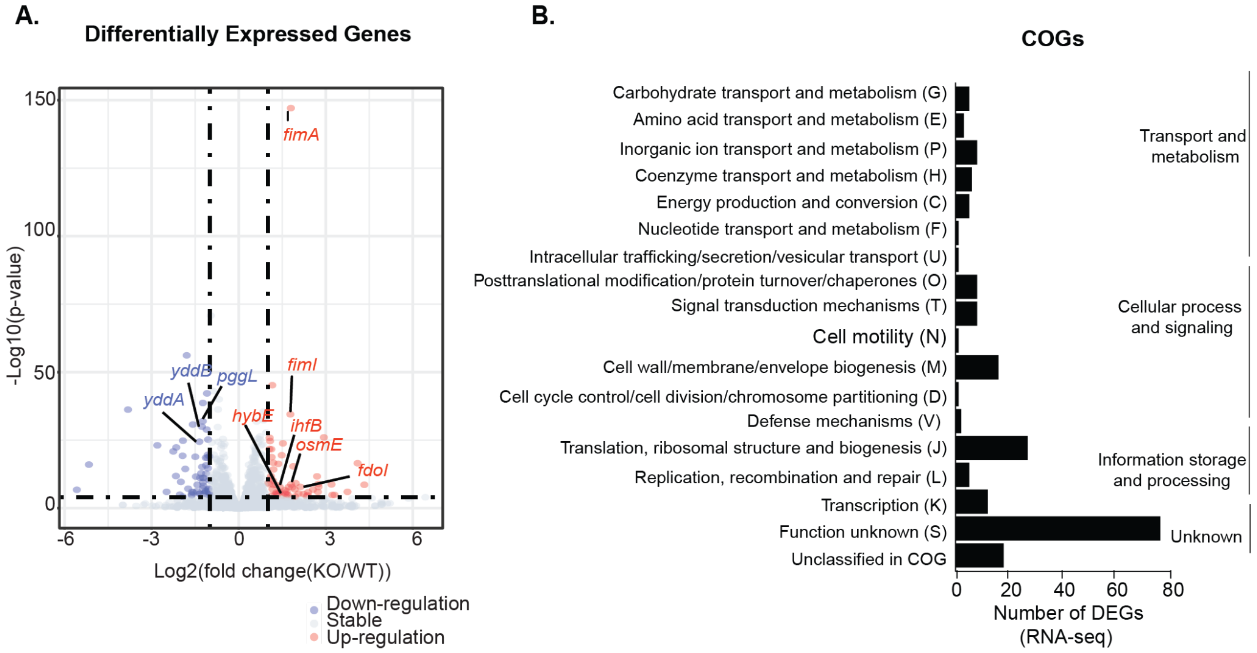

Supplementary Figure 13 Functional classification of differentially expressed genes in the *yfeC* deletion strain.

(A) 205 genes were differentially expressed after deletion of *yfeC* (cut-off value is $\log_2$ fold-change >=1, or <=-1, and adjust p-value < 0.05). (B) Functional classification of differentially expressed genes in the *yfeC* deletion strain. The top three clusters of functional groups were involved in transport and metabolism, cellular process and signaling, and information processing.

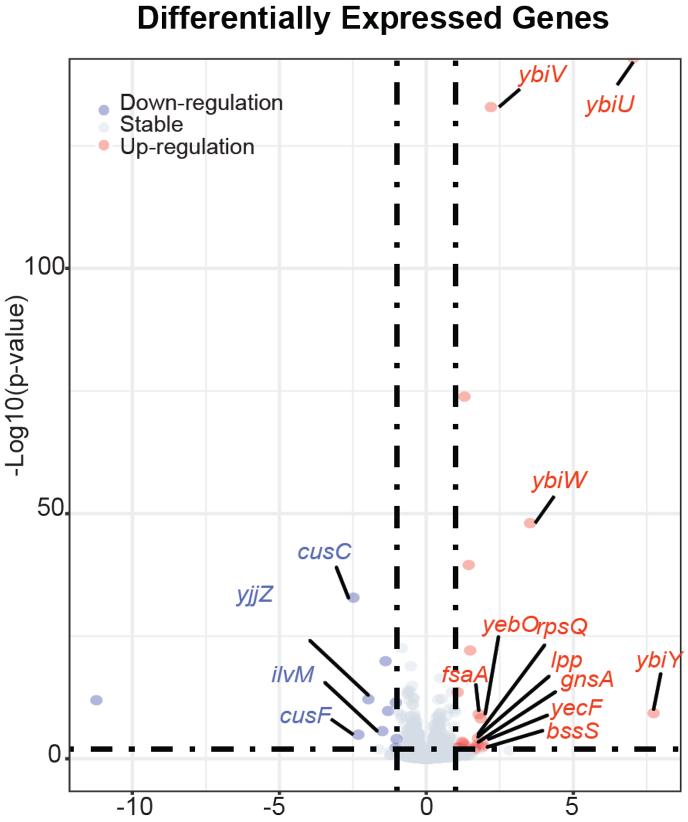

Supplementary Figure 14 The gene expression comparison between the wild type and the *yciT* deletion strains.

46 genes were differentially expressed after deletion of *yciT* (cut-off value is $\log_2$ fold-change >=1, or <=-1, and adjust p-value < 0.05).

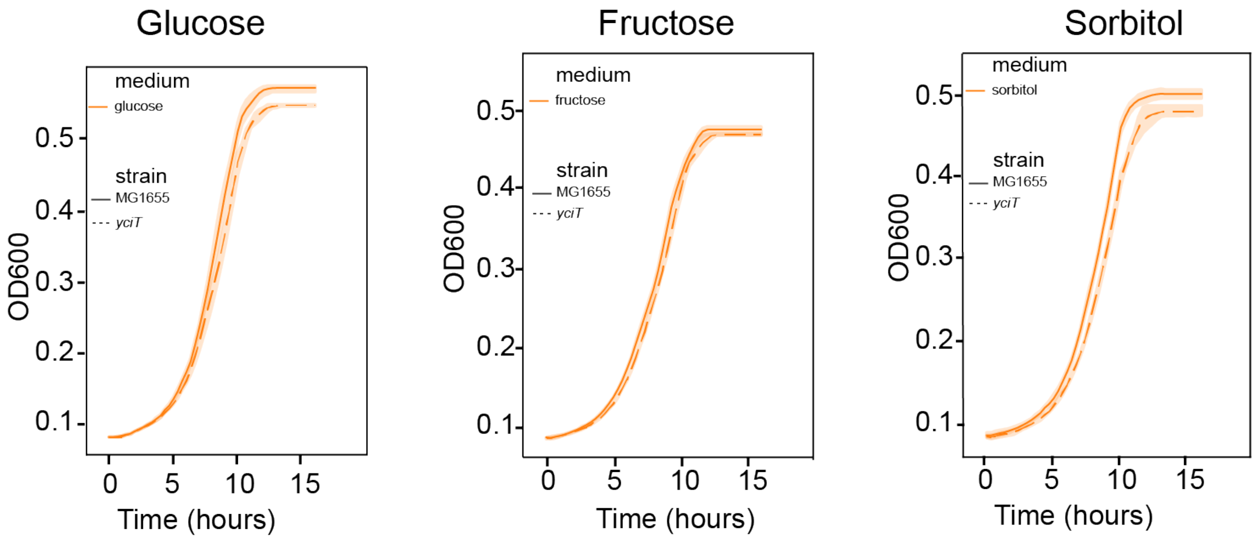

Supplementary Figure 15 The growth curve of wild type and *yciT* deletion strains at different carbon sources in the absence of osmotic stress (0.5 M NaCl). Width of shaded bands represents standard deviation of the corresponding growth trajectory.

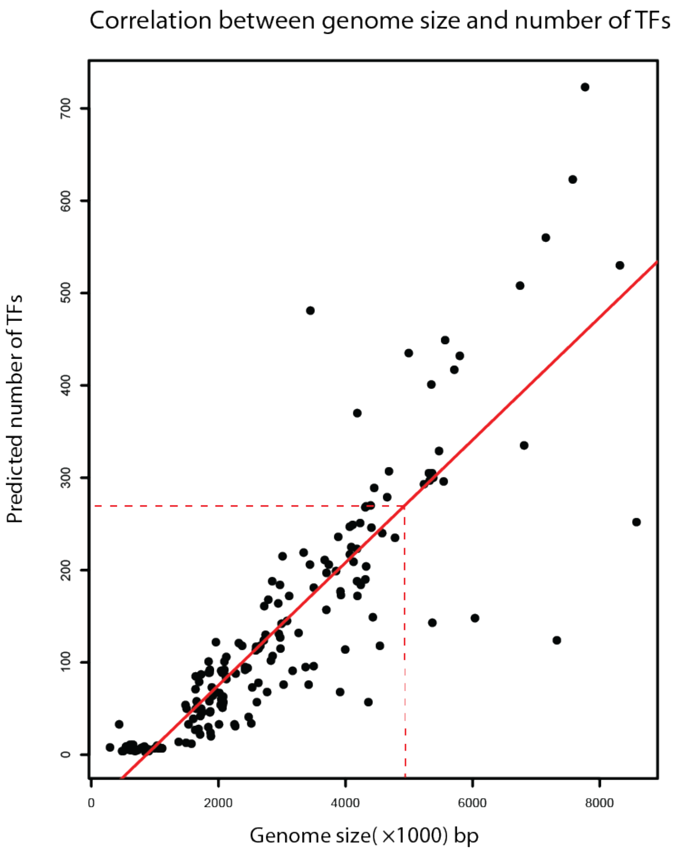

Supplementary Figure 16 The correlation between genome size and the number of TFs in the species.

The red line represents the linear correlation between them. Pearson correlation is 0.86. The red dashed line represents the relationship between *E. coli* K-12 MG1655 genome size (4.6Mbp) and estimated total number of TFs (280).

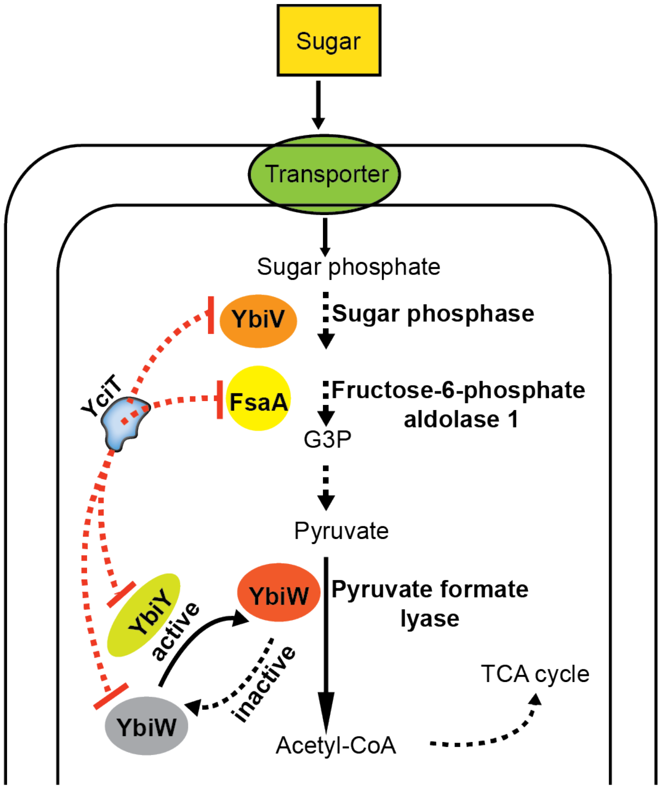

Supplementary Figure 17 The proposed pathway that is regulated by YciT.
When YciT is present (active) in *E. coli*, it represses the expression of target genes (*ybiV*, *ybiU*, *fsaA*, and *ybiY*).

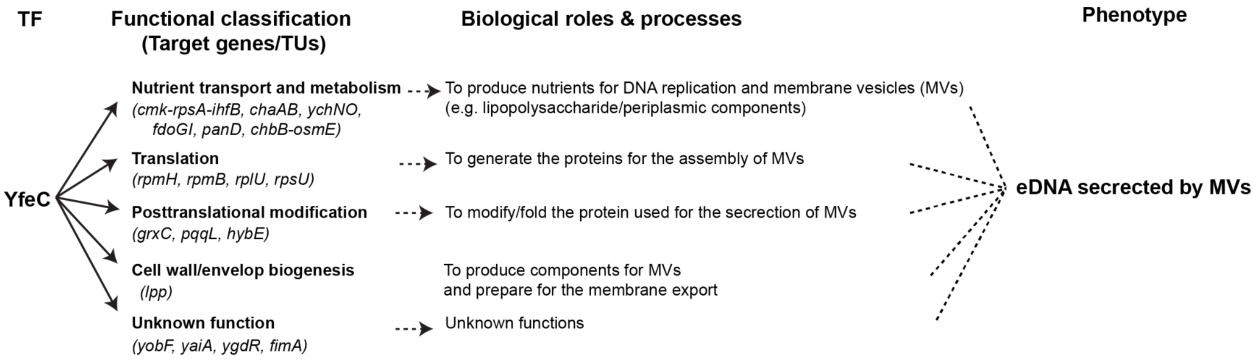

Supplementary Figure 18 YfeC regulatory networks regulate cellular functions. Arrows with solid lines indicate the regulation observed in this study. Arrows with dotted lines indicate that the regulation is not observed in this study.

Supplementary Table 1 Comparison of structure and regulatory activities between candidate TFs and known TFs in *E. coli* K-12 MG1655.

	Known TFs			Candidate TFs		
Family Type	Regulatory Role	Average size of AAs	Physiological function	Predicted regulatory Role	Average size of AAs	Representatives
LysR	Dual	Varied	Amino acid biosynthesis	Dual	~ 306	YahB, YbdO
AraC	Activator	Varied	Virulence, sugar metabolism	Repressor	~ 280	YbcM
GntR	Repressor	~ 246	Carbon metabolism	Repressor	~ 352	YdcR
TetR	Repressor	~ 210	Tetracycline resistance	Repressor	~ 210	YcfQ
LuxR	Activator	~ 220	Biosynthesis and glycerol metabolism	Activator	~ 200	YhjB
GalR/LacI	Repressor	~ 334	Carbon source uptake	Repressor	~ 332	YcjW
IclR	Repressor	~ 273	Carbon source uptake	Repressor	~ 262	YjhI
DeoR	Repressor	~ 257	Sugar metabolism	Repressor	~ 261	YihW, YciT, YgbI

N/A* indicates that there are no representative candidates for this condition.

Supplementary Table 2 Protein sequence metadata and homology modeling statistics from SWISS-MODEL

		Source: UniProt			Source: SWISS-MODEL			
**Gene ID**	**Protein name**	**UniProt**	**DNA binding domain motif**	**DNA binding domain residues**	**PDB template**	**GMQE (Global Model Quality Estimate)**	**Oligomeric state prediction**	**QMEAN Z-score**
b3711	YidZ	P31463	H-T-H motif	25 to 44	2esn.1.A	0.50	Dimer or Tetramer	-3.14
b2398	YfeC	P0AD37	N/A	N/A	2qww.1.A	0.34	Dimer	-3.17
b1284	YciT	P76034	H-T-H motif	18 to 37	2w48.1.A	0.34	Tetramer	-5.31
b2735	YgbI	P52598	H-T-H motif	20 to 39	2w48.1.A	0.36	Tetramer	-5.57
b1320	YcjW	P77615	H-T-H motif	5 to 24	1vpw.1.B	0.67	Dimer	-2.36
b1434	YdcN	P77626	H-T-H motif	23 to 42	1y9q.1.A	0.75	Dimer	-1.94

Note, N/A indicates that there is no available information in the UniProt.

Supplementary Table 3 The target genes directly regulated by YfeC

b_number	Transcriptional unit	Description/Function	COGs	log2(FoldChange)	padj
b3703	*rpmH*	50S ribosomal subunit protein L34	Translation, ribosomal structure and biogenesis	4.33079132	4.36E-08
b3637	*rpmB*	50S ribosomal subunit protein L28	Translation, ribosomal structure and biogenesis	1.15981833	0.01940833
b3186	*rplU*	50S ribosomal subunit protein L21	Translation, ribosomal structure and biogenesis	1.59875753	0.00000297
b3065	*rpsU*	30S ribosomal subunit protein S21	Translation, ribosomal structure and biogenesis	1.93796714	0.00230352
b2399	*yfeCD*	putative transcription factor	Function unknown	4.32546198	4.98E-133
b1161	*ycgFX*	blue light- and temperature-regulated antirepressor BluF	Function unknown	-1.6727558	0.01324743
b3610	*grxC*	reduced glutaredoxin 3	Post-translational modification, protein turnover, chaperones	1.65174882	0.00000821
b1494	*pqqL*	periplasmic metalloprotease	Post-translational modification, protein turnover, chaperones	-1.0556295	8.21E-24
b2992	*hybE*	hydrogenase 2-specific chaperone	Post-translational modification, protein turnover, chaperones	1.51293908	0.00021498

Supplementary Table 3 The target genes directly regulated by YfeC (con't)

b_number	Transcriptional unit	Description/Function	COGs	log2(FoldChange)	padj
b0910	*cmk-rps-ihfB*	cytidylate kinase	Nucleotide transport and metabolism	1.20143761	3.07E-09
b1216	*chaAB*	Na+/K+:H+ antiporter ChaA	Inorganic ion transport and metabolism	1.55342508	0.01017597
b1219	*ychNO*	DsrE/F sulfur relay family protein YchN	Inorganic ion transport and metabolism	-1.3343157	7.36E-12
b3894	*fdoGI*	formate dehydrogenase O subunit α	Energy production and conversion	1.16191449	0.00386399
b0131	*panD*	aspartate 1-decarboxylase proenzyme	Coenzyme transport and metabolism	-1.3441817	0.0000505
b4317	*fimAICD*	outer membrane protein; export and assembly of type 1 fimbriae	Cell wall/membrane/envelope biogenesis	1.05862358	1.91E-22
b1677	*lpp*	murein lipoprotein	Cell wall/membrane/envelope biogenesis	1.7414382	0.00345401
b1738	*chbB-osmE*	N,N'-diacetylchitobiose-specific PTS enzyme IIB component	Carbohydrate transport and metabolism	1.80868592	1.81E-09
b1824	*yobF*	small protein involved in stress response	Function unknown	2.40258368	0.00952181
b0389	*yaiA*	protein YaiA	Function unknown	1.36869395	0.015577
b2833	*ygdR*	predicted protein	Function unknown	1.53268718	0.00741777

References

1. Koita,K. and Rao,C.V. (2012) Identification and analysis of the putative pentose sugar efflux transporters in Escherichia coli. *PLoS One*, **7**, e43700.

2. Rodionova,I.A., Gao,Y., Sastry,A., Monk,J., Wong,N., Szubin,R., Lim,H., Zhang,Z., Saier,M.H. and Palsson,B. PtrR (YneJ) is a novel E. coli transcription factor regulating the putrescine stress response and glutamate utilization. 10.1101/2020.04.27.065417.
